# *microRNA‐99a‐5p* induces cellular senescence in gemcitabine‐resistant bladder cancer by targeting *SMARCD1*

**DOI:** 10.1002/1878-0261.13192

**Published:** 2022-02-28

**Authors:** Motoki Tamai, Shuichi Tatarano, Shunsuke Okamura, Wataru Fukumoto, Issei Kawakami, Yoichi Osako, Takashi Sakaguchi, Satoshi Sugita, Masaya Yonemori, Yasutoshi Yamada, Masayuki Nakagawa, Hideki Enokida, Hirofumi Yoshino

**Affiliations:** ^1^ Department of Urology Graduate School of Medical and Dental Sciences Kagoshima University Japan

**Keywords:** bladder cancer, cellular senescence, gemcitabine resistance, *miR‐99a‐5p*, *SMARCD1*

## Abstract

Patients with advanced bladder cancer are generally treated with a combination of chemotherapeutics, including gemcitabine, but the effect is limited due to acquisition of drug resistance. Thus, in this study, we investigated the mechanism of gemcitabine resistance. First, gemcitabine‐resistant cells were established and resistance confirmed *in vitro* and *in vivo*. Small RNA sequencing analyses were performed to search for miRNAs involved in gemcitabine resistance. *miR‐99a‐5p*, selected as a candidate miRNA, was downregulated compared to its parental cells. In gain‐of‐function studies, *miR‐99a‐5p* inhibited cell viabilities and restored sensitivity to gemcitabine. RNA sequencing analysis was performed to find the target gene of *miR‐99a‐5p*. *SMARCD1* was selected as a candidate gene. Dual‐luciferase reporter assays showed that *miR‐99a‐5p* directly regulated *SMARCD1*. Loss‐of‐function studies conducted with si‐RNAs revealed suppression of cell functions and restoration of gemcitabine sensitivity. *miR‐99a‐5p* overexpression and *SMARCD1* knockdown also suppressed gemcitabine‐resistant cells *in vivo*. Furthermore, β‐galactosidase staining showed that *miR‐99a‐5p* induction and *SMARCD1* suppression contributed to cellular senescence. In summary, tumor‐suppressive *miR‐99a‐5p* induced cellular senescence in gemcitabine‐resistant bladder cancer cells by targeting *SMARCD1*.

## Graphical abstract

Here, we established gemcitabine‐resistant bladder cancer (BC) cell line by stepwise exposure to gemcitabine *in vitro*. We also confirmed gemcitabine resistance in a xenograft mouse model. Our data showed downregulation of *miR‐99a‐5p* and upregulation of the *miR‐99a‐5p* target *SMARCD1* in gemcitabine‐resistant BC cells, following cellular senescence inhibition. In summary, *miR‐99a‐5p* induced cellular senescence in gemcitabine‐resistant BC by targeting *SMARCD1*.

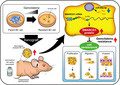

**Abbreviations**
BC: bladder cancer
BLCA: bladder urothelial carcinoma
CR: complete response
dCTP: deoxycytidine triphosphate
dFdCDP: difluorodeoxycytidine diphosphate
dFdCTP: difluorodeoxycytidine triphosphate
GEM‐R BC: gemcitabine‐resistant BC
MEM: minimum essential medium
MIBC: muscle‐invasive BC
miRNA: microRNA
NGS: next‐generation sequencer
NMIBC: nonmuscle‐invasive BC
OIS: oncogene‐induced senescence
OS: overall survival
PR: partial response
SASP: senescence‐associated secretory phenotype
TCGA: The Cancer Genome Atlas
TIS: therapy‐induced senescence

## 1 Introduction

Bladder cancer (BC) is broadly classified into nonmuscle‐invasive BC (NMIBC) and muscle‐invasive BC (MIBC) according to the depth of the cancer cells. Seventy to 80% of BC patients are diagnosed with NMIBC, and the recurrence rate is high in this group, ranging from 50% to 70%. The 5‐year survival rate for patients with MIBC is less than 50%, and about 50% of patients will have metastases within 2 years [1]. Chemotherapy with gemcitabine and cisplatin as neoadjuvant and adjuvant therapy is commonly used for patients with advanced BC [2]. This choice of chemotherapy achieves a complete response (CR) in 14.5% of patients and a partial response (PR) in 34.5%. However, the median overall survival (OS) after chemotherapy is only about 14 months [3, 4]. We have previously reported that this choice of chemotherapy for bladder cancer is enhanced by PHGDH inhibition [5]. However, the mechanism of resistance to these drugs is not fully understood and is a significant clinical problem.

microRNAs (miRNAs) are endogenous small noncoding RNA molecules of 19–22 bases in length that inhibit protein synthesis and translation [6]. In our previous studies, we have confirmed that miRNAs are involved in many functions of cancer, such as cancer cell progression, migration, and invasion [7, 8], and some miRNAs are deeply involved in human oncogenesis [9, 10]. In addition, we recently reported that miRNAs are involved in the molecular mechanism of cisplatin resistance in BC [11]. Pharmacologically, cisplatin undergoes hydrolysis and binds strongly to DNA via the N7 site of the guanine and adenine bases. This results in cross‐linking and damage to the DNA, which induces apoptosis [12, 13]. On the other hand, gemcitabine is phosphorylated by deoxycytidine kinase and converted to difluorodeoxycytidine diphosphate and triphosphate (dFdCDP and dFdCTP). dFdCTP competes with deoxycytidine triphosphate (dCTP) to inhibit the activity of DNA polymerase, while dFdCDP forms a complex with ribonucleotide reductase subunits and ATP to deplete the deoxynucleotide pool essential for DNA synthesis. The incorporation of dFdCTP into DNA is believed to be the primary mechanism that ultimately leads to cancer cell apoptosis [14]. Given these differences, we speculate that the mechanism underlying cisplatin resistance may differ from that of gemcitabine resistance. There have been several reports regarding miRNAs and gemcitabine resistance in pancreatic, breast, gallbladder, and colorectal cancers [15, 16, 17, 18]. Regarding BC, Cao et al. [19] showed that *miR‐129‐5p* inhibited gemcitabine resistance and promoted apoptosis by targeting Wnt5a, and An et al. [20] showed that expression of *miRNA‐143* enhanced gemcitabine resistance. However, the relationship between miRNAs and gemcitabine resistance is not well understood compared to other organ cancers. Therefore, in this study, we focused on the involvement of miRNAs in gemcitabine‐resistant bladder cancer.

First, we approached the mechanism of gemcitabine resistance by establishing gemcitabine‐resistant BC cell lines (GEM‐R‐BOY and GEM‐R‐T24). Next, we performed small‐RNA sequencing using the parental BC cell lines and the resistant BC cell lines to search for miRNAs associated with gemcitabine resistance. The candidate miRNAs were introduced into the gemcitabine‐resistant cell line and subjected to functional analysis. Next, the target genes of the candidate miRNAs were searched using RNA next‐generation sequencing. In addition, loss of function studies were conducted to evaluate the target genes.

## 2 Materials and methods

### 2.1 BC cell lines and cultures

**Fig. 1:**
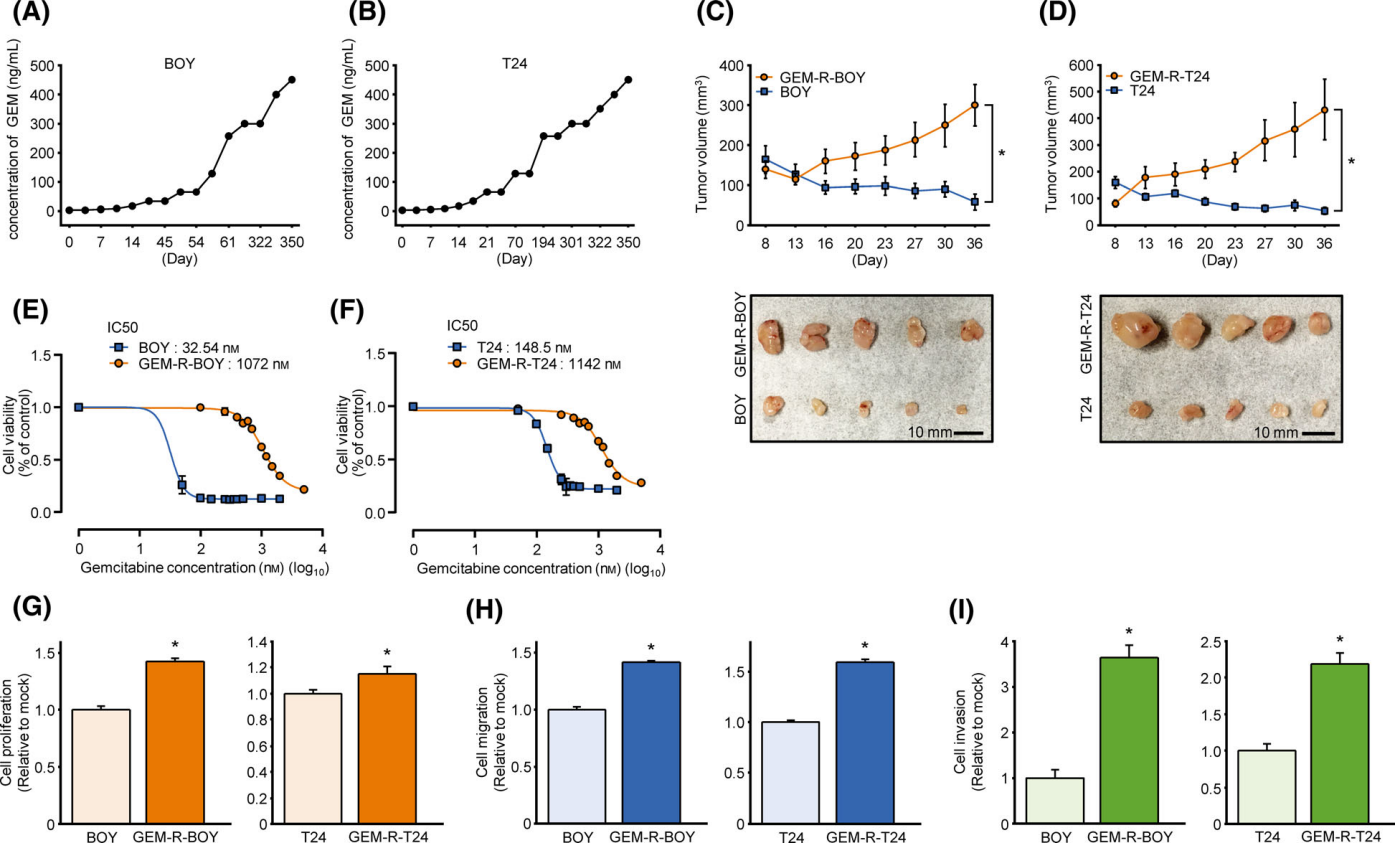
Establishment of gemcitabine‐resistant BC cell lines. (A) BOY and (B) T24 were cultured with gemcitabine at concentrations ranging from 1 to 450 µg·mL^−1^ for 12 months. Gemcitabine effects on parental and derived lines (C) BOY/GEM‐R‐BOY and (D) T24/GEM‐R‐T24. Comparison of tumor volumes after subcutaneous injection of 100 mg gemcitabine·kg^−1^ per mouse per twice a week, *n* = 5 mice per group). *P < 0.05, Mann–Whitney *U* tests. The error bars indicate SEM. Photograph shows excised tissue in the Xenograft mouse model on day 36. Scale bar, 10 mm. Calculated IC50 for parental and derived lines (E) BOY/GEM‐R‐BOY and (F) T24/GEM‐R‐T24. *n* = 6. The error bars indicate SEM. (G) Cell proliferation measured by XTT assays of parental BC and derived GEM‐R BC strains. *n* = 6. *P < 0.05, Mann–Whitney *U* tests. The error bars indicate SEM. (H) Cell migration activities of parental BC and derived GEM‐R BC strains as measured by wound healing assay. *n* = 4. *P < 0.001, Mann–Whitney *U* tests. The error bars indicate SEM. (I) Cell invasion activities of parental BC and derived GEM‐R BC strains as measured by Matrigel invasion assay. Invasion cells were counted and compared. *n* = 6. *P < 0.001, Mann–Whitney *U* tests. The error bars indicate SEM. GEM, gemcitabine; GEM‐R BC, gemcitabine‐resistant bladder cancer.

Two types of human BC cell lines were used. BOY is a cell line that was established in our laboratory and is derived from a 66‐year‐old Asian male patient diagnosed with Stage IV BC with numerous lung metastases. The cell line was performed with the understanding and written consent of the subjects and were approved by the ethics committee of the institution concerned [21]. T24 was obtained from the American Type Culture Collection (Manassas, VA, USA). See Fig. 1 for the generation of GEM‐R‐BOY and GEM‐R‐T24. These cell lines were cultured in minimum essential medium (MEM) containing 10% FBS, 50 U·mL^−1^ penicillin and 50 μg·mL^−1^ streptomycin at 37 °C in a humidified environment consisting of 95% air/5% CO_2_. To establish the GEM‐R BC cell line, the BC cell line was seeded at 300 000 per well in 35 mm dish and cultured in standard medium. Twenty‐four hours later, gemcitabine adjusted to 10 times the target concentration was mixed with 10% of the medium volume. After 48 h of continuous culture, the surviving BC cells were collected and cultured in standard medium for recovery. This process was repeated with various concentrations of gemcitabine from 1 to 450 ng·mL^−1^, and the cells were cultured for 12 months. After the establishment of the GEM‐R BC cell line, gemcitabine was continuously added to the culture medium to continue the stimulation culture [22].

### 2.2 *In vivo* validation of the GEM‐R BC cell lines

This animal experiment was approved by the Kagoshima University Animal Experiment Committee (MD20030) and were conducted in accordance with the animal licensing guidelines of the Kagoshima University Animal Care Committee. Five‐week‐old female nude mice (BALB/c‐nu/nu) purchased from Charles River Laboratories (Yokohama, Japan) were used in the study. The sample size was five per cell line and was determined based on the Guidelines for the Welfare and Use of Animals in Cancer Research [23]. Mice were kept in a rectangular mouse cage (225 × 338 × 140 mm) under standard experimental conditions (12‐h day/night cycle, temperature 25 °C). Two or three mice were housed in one cage. The cages were covered with sawdust to ensure water absorption and flexibility, and the mice were continuously fed water and a standard diet (CLEA Rodent Diet CL‐2). The cages were cleaned once a week. In the verification of gemcitabine resistance acquisition, GEM‐R BC cells (GEM‐R‐BOY, GEM‐R‐T24) and their parental BC cells (BOY, T24) were used as controls. Four BC cell lines (BOY, GEM‐R‐BOY, T24, GEM‐R‐T24) were adjusted to 5 × 10^7^ mL^−1^ and mixed with 100 µL of BC cells and 100 µL of Matrigel (BD Biosciences, Bedford, MA, USA). Two hundred microliter of parental BC cells (BOY, T24) were injected subcutaneously into the right flank and 200 µL of GEM‐R BC cells (GEM‐R‐BOY, GEM‐R‐T24) were injected into the left flank (*n* = 5). Measurements of mouse body weight and tumor size were performed twice a week, starting 8 days after inoculation. Tumor size was measured with calipers and calculated as *v* = (length × width^2^) × (π/6). Administration of gemcitabine (100 mg·kg^−1^, twice/week) [22] was also started 8 days after inoculation. Five weeks after inoculation, all mice were euthanized with 100% CO_2_ and tumor size was assessed. In the validation of *miR‐99a‐5p* and *SMARCD1*, GEM‐R BC cells (GEM‐R‐BOY, GEM‐R‐T24) were used. Using the transfection method described below, cells at a concentration of 100 000 mL^−1^ were transfected with 10 nm miRNA or small interfering RNA (siRNA) [24]. miR‐NC and *miR‐99a‐5p*, si‐NC and si‐*SMARCD1* were transfected as pairs, and cells were collected after 48 h. The BC cells were adjusted to 5 × 10^7^ mL^−1^ and mixed with 100 µL of BC cells and 100 µL of Matrigel (BD Biosciences). Two hundred microliter of control group cells (miR‐NC, si‐NC) were injected into the left flank and 200 µL of *miR‐99a‐5p* and *SMARCD1* groups cells were injected into the right flank (*n* = 5). Measurements of mouse body weight and tumor size were performed twice a week, starting after inoculation. Tumor size was measured with calipers and calculated as *v* = (length × width^2^) × (π/6). Nineteen days after inoculation, all mice were euthanized with 100% CO_2_ and tumor size was assessed. There were no criteria for inclusion or exclusion of animals during the experiment, and no data points were excluded for any experimental group. In the animal experiments, no adverse events were observed, and no confounding factors were observed. All animal experiments were performed by MT, HY, SO, WF, and IK.

### 2.3 IC50 decision

To determine the IC50 value, cells were seeded six times in 96‐well plates at a density of 3000 cells per well and treated with a series of dilute concentrations of gemcitabine. After 96 h of incubation, cell proliferation was measured with the XTT assay method described below. Inhibition data were used to calculate IC50 values using nonlinear, four‐parameter, variable slope equation software (graphpad prism ver. 8.00 for Windows; GraphPad Software, San Diego, CA, USA).

### 2.4 Transfection of mature miRNAs and small interfering RNAs

Bladder cancer cells were transfected with 10 nm miRNA or siRNA using Lipofectamine RNAiMAX transfection reagent (Thermo Fisher Scientific, Inc., Waltham, MA, USA) and Opti‐MEM (Thermo Fisher Scientific, Inc.) as previously reported [24]. Precursor miRNA (*hsa‐miR‐99a‐5p*; product ID: PM10719; Thermo Fisher Scientific, Inc.) and negative control miRNA (negative control miRNA; product ID: AM17111; Thermo Fisher Scientific, Inc.) were used for the gain‐of‐function experiments. For the loss‐of‐function experiments, *SMARCD1* siRNA (cat No. HSS185985 and HSS185986; Thermo Fisher Scientific, Inc.) and Negative Control siRNA (D‐001810‐10; Dharmacon; Horizon Discovery Group plc, Cambridge, UK) were used.

### 2.5 miRNA and mRNA sequence analysis

Total RNA extracted from BOY, GEM‐R‐BOY, T24, and GEM‐R‐T24 cell lines was subjected to miRNA sequencing performed by RIKEN GENESIS CORPORATION (Tokyo, Japan) to search for miRNAs associated with gemcitabine resistance. Samples were prepared using the TruSeq Small RNA Library Prep Kit (Illumina, Inc., San Diego, CA, USA) according to the manufacturer's protocol. The length of the library was 141‐143 bp. Sequencing was performed using a next‐generation sequencer (NGS), HiSeq 2500 (Illumina, Inc.). The effective read length was 50 bp, and the analysis was performed in Single End/Multiplex. By comparing the parental BC cells with the GEM‐R BC cell lines (BOY and GEM‐R‐BOY, T24 and GEM‐R‐T24), we selected miRNAs whose expression was significantly reduced in the GEM‐R BC cell lines (log_2_ fold‐change < −1.0). In order to identify the target mRNA of *miR‐99a‐5p*, mRNA sequence analysis was performed at RIKEN GENESIS, Inc. Samples were prepared using the TruSeq Stranded mRNA Library Prep Kit (Illumina, Inc.) according to the manufacturer's protocol. The library was prepared by adding adapters to the fragmented RNA samples. The length of the library was 303–314 bp. Sequencing of the formed clusters in the S4 flow cell was performed using NovaSeq 6000 (Illumina, Inc.), a next‐generation sequencer (NGS). The effective read length was 100 bp, and the analysis was performed using the paired‐end/multiplex method. Candidate target genes were significantly downregulated after transfection with *miR‐99a‐5p* in GEM‐R‐BOY and GEM‐R‐T24 compared to control miRNA (log_2_ fold‐change < −1.0).

### 2.6 *In silico* analysis

We evaluated the clinical relevance of our findings using the TCGA cohort database, which consists of 436 patients with bladder urothelial carcinoma (BLCA). The study followed the standards of the publication guidelines provided by TCGA. To search for miRNAs associated with gemcitabine resistance, we extracted miRNAs from NGS results that were underexpressed in GEM‐R BC cells compared to parental BC cells in both BOY and T24 and identified miRNAs that had been reported to be involved with gemcitabine in other organ cancers and merged these results. To identify potential targets of *miR‐99a‐5p*, genes reduced by transfection of *miR‐99a‐5p* were subjected to mRNA sequence analysis based on TargetScan database Release 7.2 (http://www.targetscan.org).

### 2.7 RNA extraction and RT‐qPCR

For the extraction of total RNA, cultured cells were lysed in ISOGEN (Nippon Gene, Tokyo, Japan) and the manufacturer's protocol was followed. RNA concentration was measured spectrophotometrically, and RNA quality was checked with an Agilent 2100 Bioanalyzer (Agilent Technologies, Santa Clara, CA, USA). To quantify the expression level of *miR‐99a‐5p*, stem‐loop RT‐PCR (TaqMan miRNA Assays; P/N: 4427975 for *miR‐99a‐5p*; Applied Biosystems; Thermo Fisher Scientific, Inc.) was used according to previously reported conditions [24]. For *SMARCD1*, the SYBR green quantitative PCR‐based array approach was applied. The primer sets used to measure the mRNA expression levels of *SMARCD1* were as follows: forward primer, 5′‐AAACGGAAGCTGCGAATTTTC‐3′ and reverse primer, 5′‐AGCCGTCCTTCTACCCGAA‐3′. For the endogenous control, beta‐glucuronidase (GUSB) was used. The set consisted of forward primer, 5′‐CGTCCCACCTAGAATCTGCT‐3′ and reverse primer, 5′‐TTGCTCACAAAGGTCACAGG‐3′. The specificity of the amplification was monitored using the dissociation curve of the amplified product. Gene expression levels relative to GUSB were calculated by 2^−ΔΔCT^ method.

### 2.8 Western blotting

NuPAGE LDS Sample Buffer (Invitrogen; Thermo Fisher Scientific, Inc.) was used for the preparation of total protein lysates. The following antibodies were used for immunoblotting: anti‐SMARCD1 (1 : 500; cat. no. A301‐595A; Bethyl Laboratories, Inc., Montgomery, TX, USA), anti‐cleaved PARP (1 : 1000, #5625; Cell Signaling Technology, Inc., Danvers, MA, USA), anti‐PARP (1 : 1000, #9532; Cell Signaling Technology), anti‐β‐actin (1 : 5000; cat. no. bs‐0061R; Bios, Beijing, China), and anti‐p21^waf1/cip1^ (1 : 500, #2947; Cell Signaling Technology). Secondary antibodies were either peroxidase‐conjugated anti‐rabbit IgG (1 : 5000; cat. no. 7074S; Cell Signaling Technology, Inc.) or anti‐mouse IgG (1 : 5000; cat. no. 7074S; Cell Signaling Technology, Inc.). Protein levels were assessed using imagej software (ver. 1.52; http://rsbweb.nih.gov/ij/index.html) using the methods described previously [25, 26].

### 2.9 Assays for cell proliferation, migration, invasion, and β‐galactosidase staining, apoptosis

For the evaluation of cell proliferation, the XTT assay was used. T24 and BOY cells were seeded six times in 96‐well plates at a density of 3000 cells per well with 100 μL of medium containing 10% FBS. Ninety‐six hour after seeding, the degree of cell proliferation was measured as described above using Cell Proliferation Kit II (Roche Diagnostics GmbH, Mannheim, Germany). When gemcitabine was used, 10 μL was added, adjusted to 10 times the target concentration, and the final medium volume was adjusted to 100 μL per well. For cell migration activity, the wound healing assay was used. Cells (2.5 × 10^5^ per well) were plated in 6‐well plates and cultured for 48 h. Then, the cell monolayer was scraped using a P‐1000 micropipette tip. The initial gap length (0 h) and the remaining gap length after 12 h were calculated from micrographs. Four random microscopic fields were used for quantification. For the cell invasion assay, a BioCoat Matrigel invasion chamber consisting of a cell culture insert with a PET membrane with a pore size of 8.0 μm coated with a thin layer of Matrigel basement membrane matrix was used in a 24‐well tissue culture companion plate (CORNING, Bedford, MA, USA). Cells that passed through the pores and adhered to the surface of the chamber after 24 h were counted from micrographs. Six random microscopic fields were used for quantification. The β‐galactosidase staining assay was performed using a Senescence β‐Galactosidase Staining Kit (#9860; Cell Signaling Technology, Inc.). After treating the cells with fixative solution, pH 6.0, according to the manufacturer's protocol, the Staining Solution mixture containing X‐Gal was added, and the cells were stained overnight in a dry incubator without CO_2_. For quantification, imagej software (ver. 1.52; http://rsbweb.nih.gov/ij/index.html) was used to binarize and count the staining. Six random microscopic fields were used for quantification. The apoptosis assay was performed by double staining with FITC‐Annexin V and propidium iodide using FITC‐Annexin V Apoptosis Detection Kit (BD Biosciences, Franklin Lakes, NJ, USA) and flow cytometry (CytoFLEX Analyzer; Beckman Coulter, Brea, CA, USA). cyexpert 2.3 software (Beckman Coulter) was used to classify the cells into four categories: viable cells, dead cells, early apoptotic cells, and apoptotic cells. The percentages of early apoptotic and apoptotic cells were compared. Two microgram per milliliter cycloheximide‐treated cells were used as a positive control. Each experiment was repeated at least three times.

### 2.10 Plasmid construction and dual‐luciferase reporter assay

A partial wild‐type (WT) sequence of the 3′‐UTR of *SMARCD1* or a sequence with the *miR‐99a‐5p* target site deleted was inserted between the XhoI and PmeI restriction sites of the 3′‐UTR of the hRluc gene in the psiCHECK‐2 vector (C8021; Promega, Madison, WI, USA). GEM‐R‐BOY and GEM‐R‐T24 cells were transfected with 50 ng of vector and 10 nm of *miR‐99a‐5p*. Following the manufacturer's protocol (E1960; Promega), the activity of firefly and *Renilla* luciferase in cell lysates was measured using a dual‐luciferase assay system.

### 2.11 Immunohistochemistry

Immunohistochemistry was performed using an UltraVision Detection System (Thermo Scientific, Fremont, CA, USA) according to the manufacturer's instructions. The primary rabbit monoclonal antibodies against Ki67 (ab92742; Abcam, Cambridge, UK) were diluted 1 : 500 and incubated at 4 °C overnight. The secondary antibody was Goat Anti‐Rabbit IgG Antibody (H + L), Biotinylated (BA‐1000; Vector Laboratories, San Francisco, CA, USA) diluted to 5 µg·mL^−1^ and incubated for 30 min. Positive cells were quantitated by counting six random microscopic fields. These experimental procedures were described in a previous report [27].

### 2.12 Statistical analysis

The relationships between two groups were analyzed using Mann–Whitney *U* tests. The relationships between three or more groups were analyzed using the multiple comparison test with the Bonferroni/Dun method. Spearman's rank tests were used to evaluate the correlations between the expression of *miR‐99a‐5p* and the expression of *SMARCD1*. All analyses were using expert statview software, version 5.0 (SAS Institute, Inc., Cary, NC, USA). A *P* value of less than 0.05 was accepted as statistically significant.

### 2.13 Ethics approval and consent to participate

This work was conducted in accordance with the Declaration of Helsinki. Animal studies were approved by the Kagoshima University Animal Experiment Committee (MD20030) and were conducted in accordance with the animal licensing guidelines of the Kagoshima University Animal Care Committee. The clinical data of the patients in this study were obtained from The Cancer Genome Atlas (TCGA). TCGA is cancer genomic public data and we have not obtained individual ethical approval or written informed consent.

## 3 Results

### 3.1 Establishment of gemcitabine‐resistant BC cell lines

First, gemcitabine‐resistant BC cell lines (GEM‐R‐BOY, GEM‐R‐T24) were established. BC cells (BOY, T24) were cultured with a wide range of concentrations of gemcitabine (1–450 ng·mL^−1^) (Fig. 1A,B). GEM‐R BC cells were continuously exposed to gemcitabine to maintain tolerance. To verify drug resistance *in vivo*, we injected GEM‐R BC cells subcutaneously into the left flank of nude mice, and parental BC cells were injected subcutaneously into the right flank for comparison. Mice were intraperitoneally injected with gemcitabine twice a week [22]. Tumors of the parental BC cell xenografts (BOY and T24) showed shrinkage with intraperitoneal administration of gemcitabine, whereas tumors of GEM‐R BC xenografts (GEM‐R‐BOY and GEM‐R‐T24) were not inhibited and increased. This reflected their *in vitro* resistance to gemcitabine (Fig. 1C,D). To determine the concentration of gemcitabine resistance, IC50 values were assessed. In the case of BOY, the IC50 of GEM‐R‐BOY was more than 30 times greater than that for BOY (BOY IC50: 32.54 nm; GEM‐R‐BOY IC50: 1072 nm). For GEM‐R‐T24, it was more than seven times higher than that for T24 (T24 IC50: 148.5 nm; GEM‐R‐T24 IC50: 1142 nm) (Fig. 1E,F). The established GEM‐R BC cells showed significant differences in cell proliferation, migration, and invasion abilities compared to the parental BC cells (Fig. 1G–I, Fig. S1A,B).

### 3.2 Expression level of *miR‐99a‐5p* and its restorative effect on GEM‐R BC cell lines and BC specimens

**Fig. 2:**
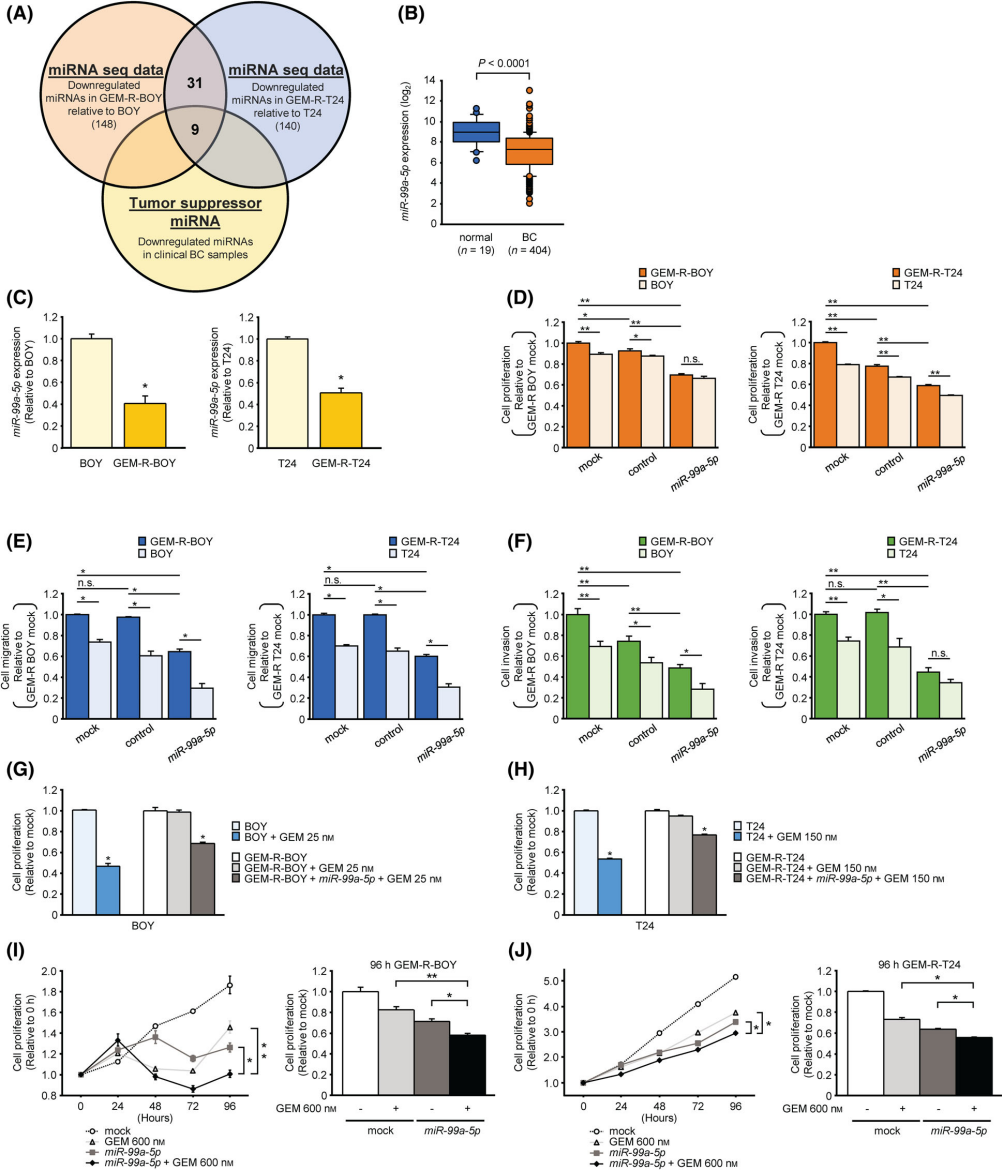
Expression level of *miR‐99a‐5p* and its restorative effect on GEM‐R BC cell lines and BC specimens. (A) A Venn diagram and *in silico* analysis of the miRNA sequences showed nine putative candidate miRNAs. (B) miRNA expression in the BLCA cohort of TCGA. *miR‐99a‐5p* expression in BLCA samples (*n* = 404) was compared with those in normal samples (*n* = 19). *P* < 0.0001, Mann–Whitney *U* tests. (C) The expression level of *miR‐99a‐5p*, as determined by qRT‐PCR, was significantly lower in the GEM‐R BC cell line than in the parental BC cell line. *n* = 3. *P < 0.001. The error bars indicate SEM. (D) Cell proliferation measured by XTT assay. *n* = 6. *P < 0.05; **P < 0.0001; ns, not significant. The error bars indicate SEM. (E) Cell migration activity measured by wound healing assay. *n* = 4. *P < 0.0001; ns, not significant. The error bars indicate SEM. (F) Cell invasion activity measured by Matrigel invasion assay. Invasion cells were counted and compared. *n* = 6. *P < 0.05; **P < 0.01; ns, not significant. The error bars indicate SEM. Transfection of *miR‐99a‐5p* increases the sensitivity of GEM‐R‐BC cell line to GEM. Each IC50 concentration of gemcitabine was given to parental cells and gemcitabine‐resistant cells. Transfection of 10 nm *miR‐99a‐5p* enhanced gemcitabine sensitivity of (G) GEM‐R‐BOY cells and (H) GEM‐R‐T24 cells by XTT assay. *n* = 6. *P < 0.0001. The error bars indicate SEM. Transfection of *miR‐99a‐5p* and administration of gemcitabine had a clear additive effect in (I) GEM‐R‐BOY and (J) GEM‐R‐T24 cells, significantly inhibiting cell proliferation. *n* = 6. *P < 0.01; **P < 0.0001. The error bars indicate SEM. The relationships between two groups were analyzed using Mann–Whitney *U* tests. The relationships between three or more groups were analyzed using the multiple comparison test with the Bonferroni/Dun method. These experiments were repeated at least three times. GEM, gemcitabine; GEM‐R BC, gemcitabine‐resistant bladder cancer; miRNA, microRNA; BLCA, bladder urothelial carcinoma.

To search for miRNAs associated with gemcitabine resistance, we performed miRNA sequencing of the parental BC cell line and the GEM‐R BC cell line. Thirty‐one miRNAs in total were downregulated in both the GEM‐R‐BOY and GEM‐R‐T24 cell lines (Fig. 2A). Using the dataset reported by Itesako et al. [28], we searched for tumor‐regulating miRNAs while comparing BC and normal bladder epithelium. In the end, nine miRNAs were identified as candidates (Fig. 2A, Table 1). Within the TCGA bladder urothelial carcinoma (BLCA) cohort, *miR‐99a‐5p* was significantly downregulated compared to normal tissues (Fig. 2B). In this study, we focused on *miR‐99a‐5p* as a promising candidate tumor suppressor involved in gemcitabine resistance. Moreover, it had also been reported to be involved in drug resistance in other organ cancers [29].

**Table 1:** Potential miRNAs.

miRNA	Log_2_ (fold change) Parent vs. GEM‐R	
	BOY	T24
*hsa‐miR‐99a‐5p*	−1.34471	−1.27743
*hsa‐let‐7c‐5p*	−1.19795	−1.01731
*hsa‐miR‐1249*	−1.00344	−1.10821
*hsa‐miR‐125b‐2‐3p*	−1.72960	−1.03480
*hsa‐miR‐137*	−1.38462	−1.18050
*hsa‐miR‐142‐5p*	−1.88532	−1.23208
*hsa‐miR‐153‐3p*	−1.12077	−1.70190
*hsa‐miR‐2355‐5p*	−1.04134	−1.32414
*hsa‐miR‐3130‐5p*	−2.12304	−1.05306

First, we confirmed by qRT‐PCR that the expression of *miR‐99a‐5p* was decreased in GEM‐R BC cells compared to the parental BC cells (Fig. 2C). Moreover, qRT‐PCR confirmed the gain of function of *miR‐99a‐5p* in parental BC cell lines (BOY and T24) and GEM‐R BC cell lines (GEM‐R‐BOY and GEM‐R‐T24) that had been transfected with *miR‐99a‐5p* (Fig. S2A). *miR‐99a‐5p*‐transfected parental BC cells and GEM‐R BC cells were significantly inhibited in their proliferation compared to cells transfected with mock or miRNA control in XTT assays (Fig. 2D, Fig. S2B). In addition, cell migration activity in the wound healing assay and cell invasion ability in the Matrigel invasion assay were also significantly suppressed in cells transfected with *miR‐99a‐5p* compared to the comparable control cells (Fig. 2E,F, Fig. S2C,D).

We further examined whether transfection of *miR‐99a‐5p* was associated with gemcitabine sensitivity in the GEM‐R BC cell line. In GEM‐R‐BOY, treatment with 25 nm gemcitabine (close to the IC50 concentration of BOY) did not inhibit cell viability, but treatment with 25 nm gemcitabine after transfection with *miR‐99a‐5p* decreased cell viability. This suggests that the sensitivity level of GEM‐R‐BOY to gemcitabine was enhanced by transfection with *miR‐99a‐5p* (Fig. 2G). Similar results were obtained for T24 cells at 150 nm gemcitabine (Fig. 2H). Next, we compared cell proliferation when gemcitabine treatment was combined with transfection of *miR‐99a‐5p*. Based upon the IC50 of GEM‐R‐BOY, we administered gemcitabine at a high concentration of 600 nm. The combination of *miR‐99a‐5p* transfection and gemcitabine treatment resulted in an additive synergistic effect, significantly inhibiting cell proliferation (Fig. 2I). Similar results were obtained for GEM‐R‐T24 (Fig. 2J). These results suggested that *miR‐99a‐5p* functions as a tumor suppressor in GEM‐R cells and enhances their sensitivity to gemcitabine.

### 3.3 Identification of *SMARCD1* mRNA as a target regulated by *miR‐99a‐5p* in the GEM‐R BC cell line

**Fig. 3:**
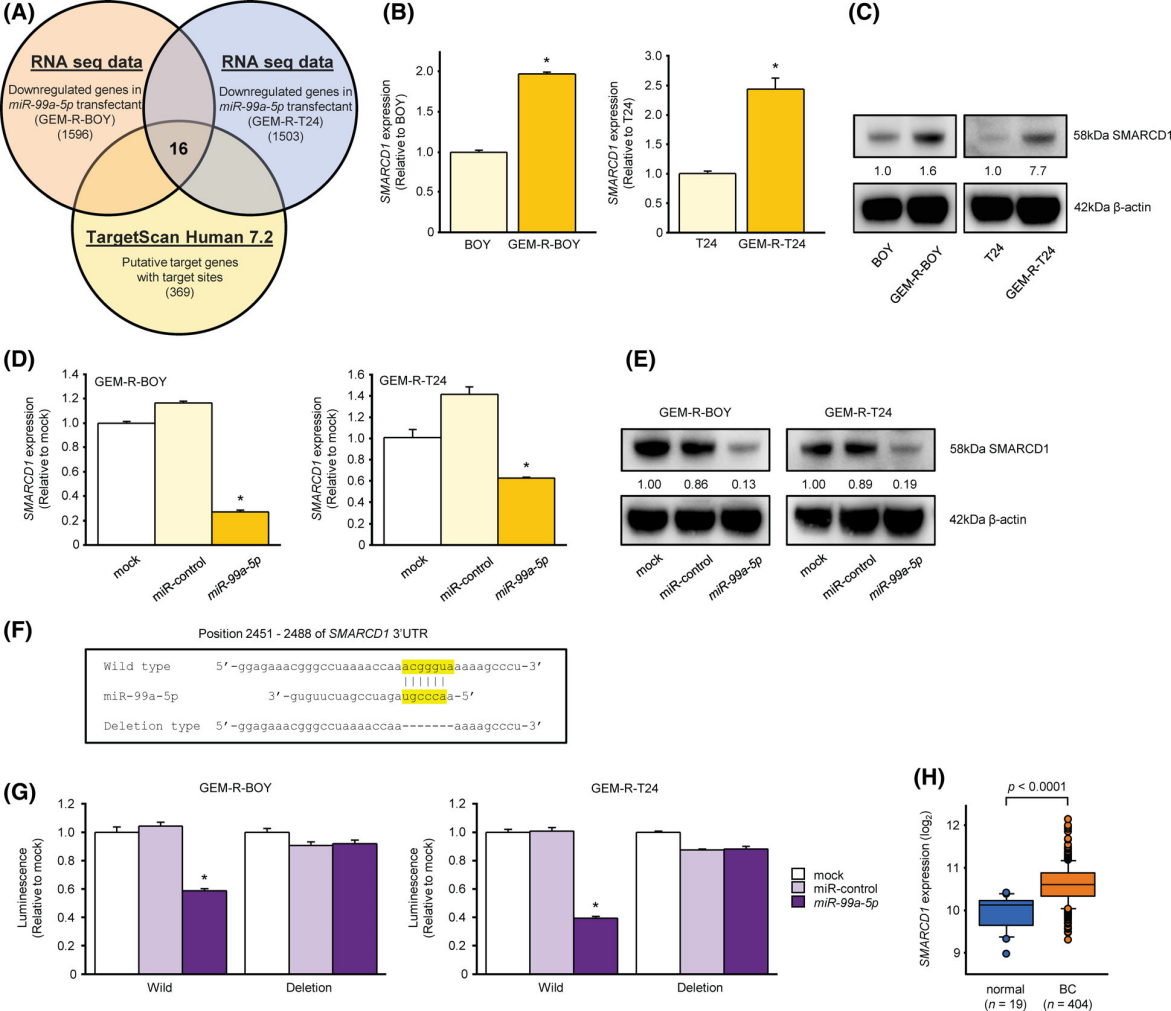
Identification of *SMARCD1* mRNA as a target regulated by *miR‐99a‐5p* in the GEM‐R‐BC cell line. (A) Venn diagram and *in silico* analysis of mRNA sequences showed that 16 putative target candidate genes of *miR‐99a‐5p* are key molecules for gemcitabine‐resistant BC. (B) The mRNA expression level of *SMARCD1* in the parental BC and GEM‐R BC strains was measured by qRT‐PCR and (C) the protein expression level was measured by western blot. The expression level of *SMARCD1* was significantly higher in the GEM‐R BC strain. *n* = 3. *P < 0.0001, Mann–Whitney *U* tests. The error bars indicate SEM. imagej was used for protein levels. (D) mRNA expression of *SMARCD1* in *miR‐99a‐5p* transfectants was measured by qRT‐PCR and (E) protein expression was measured by western blot. The expression of *SMARCD1* was lower than that in mock or miRNA control transfectants. *n* = 3. *P < 0.0001, Bonferroni/Dun method. The error bars indicate SEM. imagej was used for protein levels. (F) Presumed miRNA target sites in WT or deleted regions. (G) Dual‐luciferase reporter assay using vectors encoding putative miRNA target sites in WT or deleted regions. The luminescence intensity was significantly reduced by cotransfection of *miR‐99a‐5p* and a vector with the 3′‐UTR of WT. *n* = 3. *P < 0.0001, Bonferroni/Dun method. The error bars indicate SEM. (H) *SMARCD1* mRNA expression in the BLCA cohort of TCGA. *SMARCD1* expression in BLCA samples (*n* = 404) was compared with that in normal samples (*n* = 19). *P* < 0.0001, Mann–Whitney *U* tests. These experiments were repeated at least three times. GEM‐R BC, gemcitabine‐resistant bladder cancer; BLCA, bladder urothelial carcinoma; miRNA, microRNA; WT, wild‐type.

Next, we sought further insight into the molecular mechanisms regulated by the tumor suppressor *miR‐99a‐5p*. We used a combination of *in silico* and RNA sequencing analysis to search for genes that might be targets of *miR‐99a‐5p* in GEM‐R BC cells. TargetScan database Release 7.2 (http://www.targetscan.org) showed that *miR‐99a‐5p* might target 369 mRNAs. Next, the number of genes was narrowed down based on the expression profiles of mRNAs before and after transfection of *miR‐99a‐5p* into the GEM‐R BC cell line. Finally, 16 candidate target genes (*CLDN11*, *CTDSPL*, *CXCL16*, *FGFR3*, *IFIT2*, *NIPAL2*, *PPM1H*, *RRN3*, *SLC44A1*, *SMARCD1*, *SRMS*, *ST6GALNAC4*, *SUDS3*, *TMEM30A*, *TTC39A*, *ZNF19*) were selected (Fig. 3A, Table 2). Among them, the expression of *SMARCD1* gene was commonly upregulated in GEM‐R BC cells compared to parental BC cells (Fig. 3B, Fig. S3A). Furthermore, the expression level of *SMARCD1* mRNA was consistently decreased in cells transfected with *miR‐99a‐5p* (Fig. 3D, Fig. S3B). Similar results were obtained for the corresponding protein (Fig. 3C,E). We performed a dual‐luciferase reporter assay using the GEM‐R BC cell line to investigate whether *SMARCD1* was directly regulated by *miR‐99a‐5p*. *miR‐99a‐5p* is predicted to have one binding site according to the TargetScan database (Fig. 3F). A vector encoding a partial WT sequence of the 3′‐UTR of *SMARCD1* containing the target site of *miR‐99a‐5p* was used (Fig. 3F). The luminescence intensity was significantly reduced by co‐transfection of the vector with *miR‐99a‐5p* and the WT 3′‐UTR, but not by transfection in the deletion vector lacking the binding site (Fig. 3G). These data suggest that *miR‐99a‐5p* bound directly to the specific sequence of the 3′‐UTR of *SMARCD1* mRNA.

**Table 2:** Potential target genes.

Gene	Log_2_ (fold change) mock vs. *miR‐99a‐5p* transfectant	
	GEM‐R‐BOY	GEM‐R‐T24
*SMARCD1*	−1.14615	−1.14187
*CLDN11*	−2.22391	−1.26722
*CTDSPL*	−2.05276	−1.10187
*CXCL16*	−1.98434	−2.23436
*FGFR3*	−1.78969	−1.63200
*IFIT2*	−1.62194	−1.28213
*NIPAL2*	−1.33026	−2.13701
*PPM1H*	−1.27607	−1.30889
*RRN3*	−1.19377	−1.72054
*SLC44A1*	−1.15659	−1.26833
*SRMS*	−1.11791	−1.13572
*ST6GALNAC4*	−1.11315	−1.36084
*SUDS3*	−1.10332	−1.43725
*TMEM30A*	−1.05616	−1.35897
*TTC39A*	−1.03850	−1.22236
*ZNF19*	−1.00898	−1.39787

We next examined the relationship between *SMARCD1* expression levels in the TCGA cohort: *SMARCD1* expression was significantly elevated in bladder cancer specimens compared to adjacent noncancerous tissues (*P* < 0.0001; Fig. 3H). However, when we selected BC patients who had received gemcitabine‐based chemotherapy and compared the expression levels of *SMARCD1* in the gemcitabine‐treated and nontreated groups, we found lower expression in the treated group (Fig. S3C). We also compared cohorts of several patient groups who had received gemcitabine and found no significant difference (Fig. S3D). This was also true for the expression level of *miR‐99a‐5p* (Fig. S3E,F), but it is difficult to compare the clinical data of gemcitabine treatment with the pure gemcitabine monotherapy group or to use the therapeutic judgment data of that treatment, so it is assumed that significant data could not be extracted. On the other hand, in the scatter plot of *miR‐99a‐5p* and *SMARCD1* expression levels in the clinical data, data with a correlation trend similar to the present experiment were extracted (Fig. S3G).

### 3.4 Effect of *SMARCD1* knockdown in GEM‐R BC cells

**Fig. 4:**
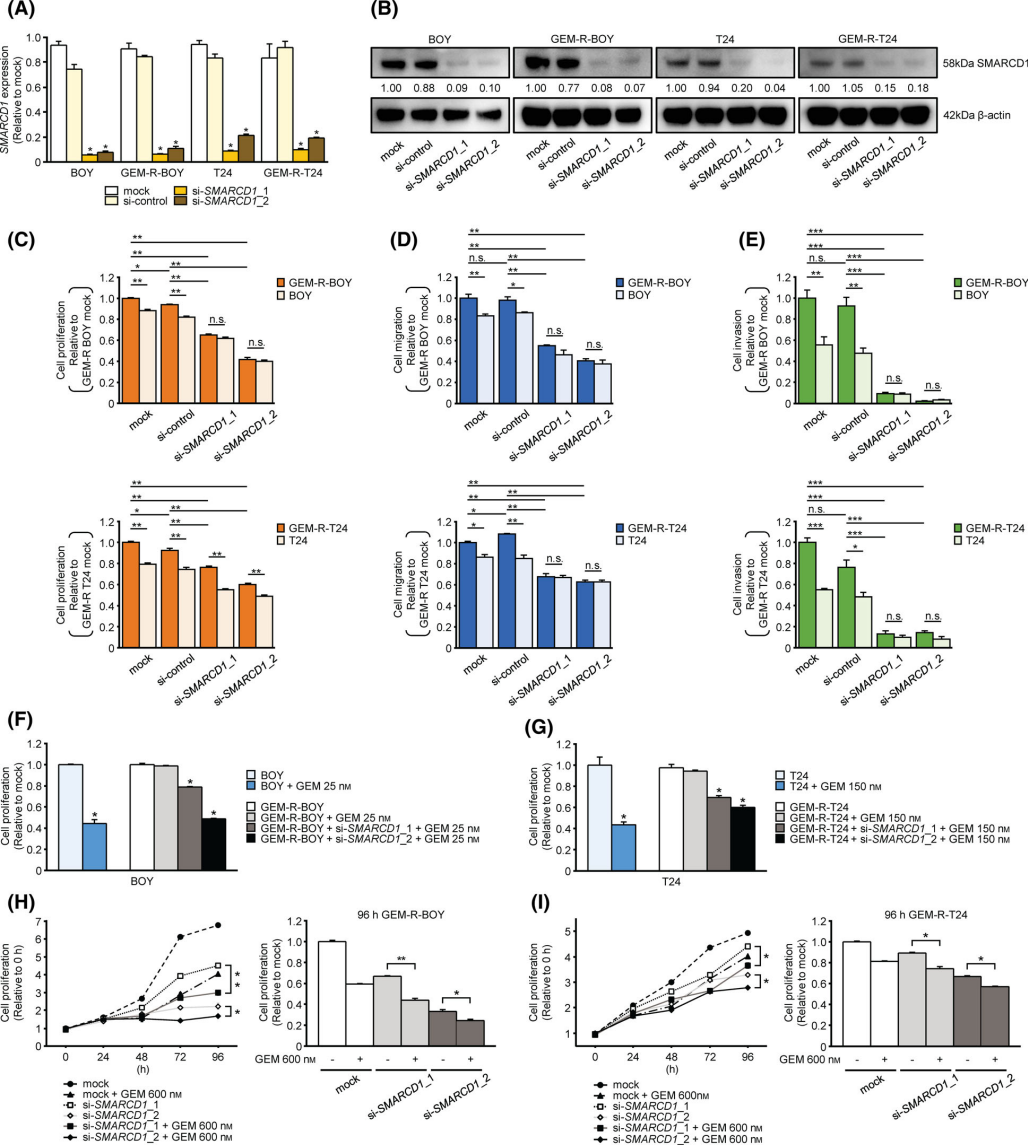
Effect of *SMARCD1* knockdown in GEM‐R BC cells. The knockdown efficiency of si‐*SMARCD1* was verified by evaluating (A) the expression level of *SMARCD1* mRNA measured by RT‐qPCR and (B) the SMARCD1 protein level measured by western blot analysis. *n* = 3. *P < 0.0001. The error bars indicate SEM. imagej was used for protein levels. (C) Cell proliferation by XTT assay. *n* = 6. *P < 0.01; **P < 0.0001; ns, not significant. The error bars indicate SEM. (D) Cell migration activity measured by wound healing assay. *n* = 4. *P < 0.01; **P < 0.0001; ns, not significant. The error bars indicate SEM. (E) Cell invasion activity measured by Matrigel invasion assay. Invasion cells were counted and compared. *n* = 6. *P < 0.05; **P < 0.01; ***P < 0.0001; ns, not significant. The error bars indicate SEM. All experiments were performed in quadruplicate and si‐*SMARCD1* transfectants were compared with mock or si‐control transfectants. Transfection of si‐*SMARCD1* increased the sensitivity of the GEM‐R BC cell line to gemcitabine. IC50 concentrations of gemcitabine were given to BC cells. Ten nanomolar si‐*SMARCD1* transfection increased gemcitabine sensitivity in (F) GEM‐R‐BOY cells and (G) GEM‐R‐T24 cells, as determined by XTT assay. *n* = 6. *P < 0.0001. The error bars indicate SEM. The combination of si‐*SMARCD1* transfection and gemcitabine administration had a clear additive effect on (H) GEM‐R‐BOY cells and (I) GEM‐R‐T24 cells and significantly inhibited cell proliferation. *n* = 6. *P < 0.001; **P < 0.0001. The error bars indicate SEM. The relationships between two groups were analyzed using Mann–Whitney *U* tests. The relationships between three or more groups were analyzed using the multiple comparison test with the Bonferroni/Dun method. These experiments were repeated at least three times. GEM, gemcitabine; GEM‐R BC, gemcitabine‐resistant bladder cancer.

To investigate the functional role of *SMARCD1* in GEM‐R BC cells, loss‐of‐function assays were performed using si‐*SMARCD1* transfection. Two types of si‐*SMARCD1* significantly reduced the expression of *SMARCD1* mRNA and protein (Fig. 4A,B). The XTT assay showed that cell proliferation was significantly inhibited in both of the parental BC cell lines and the GEM‐R BC cell lines that were transfected with si‐*SMARCD1* compared to cells transfected with mock or si‐control (Fig. 4C, Fig. S4A). Furthermore, in Matrigel invasion assays and wound healing assays, transfection of GEM‐R BC cells with si‐*SMARCD1* significantly inhibited the invasion and migration ability of the cells compared to control cells (Fig. 4D,E, Fig. S4B,C).

The proliferation of GEM‐R‐BOY cells was not inhibited by 25 nm gemcitabine, but the combination of si‐*SMARCD1* transfection and gemcitabine had a clear additive effect, and cell proliferation was significantly inhibited by 25 nm gemcitabine (Fig. 4F). The same result was obtained with 150 nm gemcitabine in GEM‐R‐T24 cells (Fig. 4G). Next, in a time course, we observed the proliferative activity of GEM‐R BC cells using combined treatment with a high concentration of gemcitabine (600 nm) and si‐*SMARCD1* transfection. We found that the combined treatment further inhibited cell proliferation compared to the treatment with each factor alone (Fig. 4H,I). These results suggest that *SMARCD1* is involved in BC's gemcitabine resistance and that inhibition of *SMARCD1* may improve the sensitivity of cancer cells to gemcitabine.

### 3.5 Overexpression of *miR‐99a‐5p* and knockdown of *SMARCD1* inhibit tumor growth *in vivo*

**Fig. 5:**
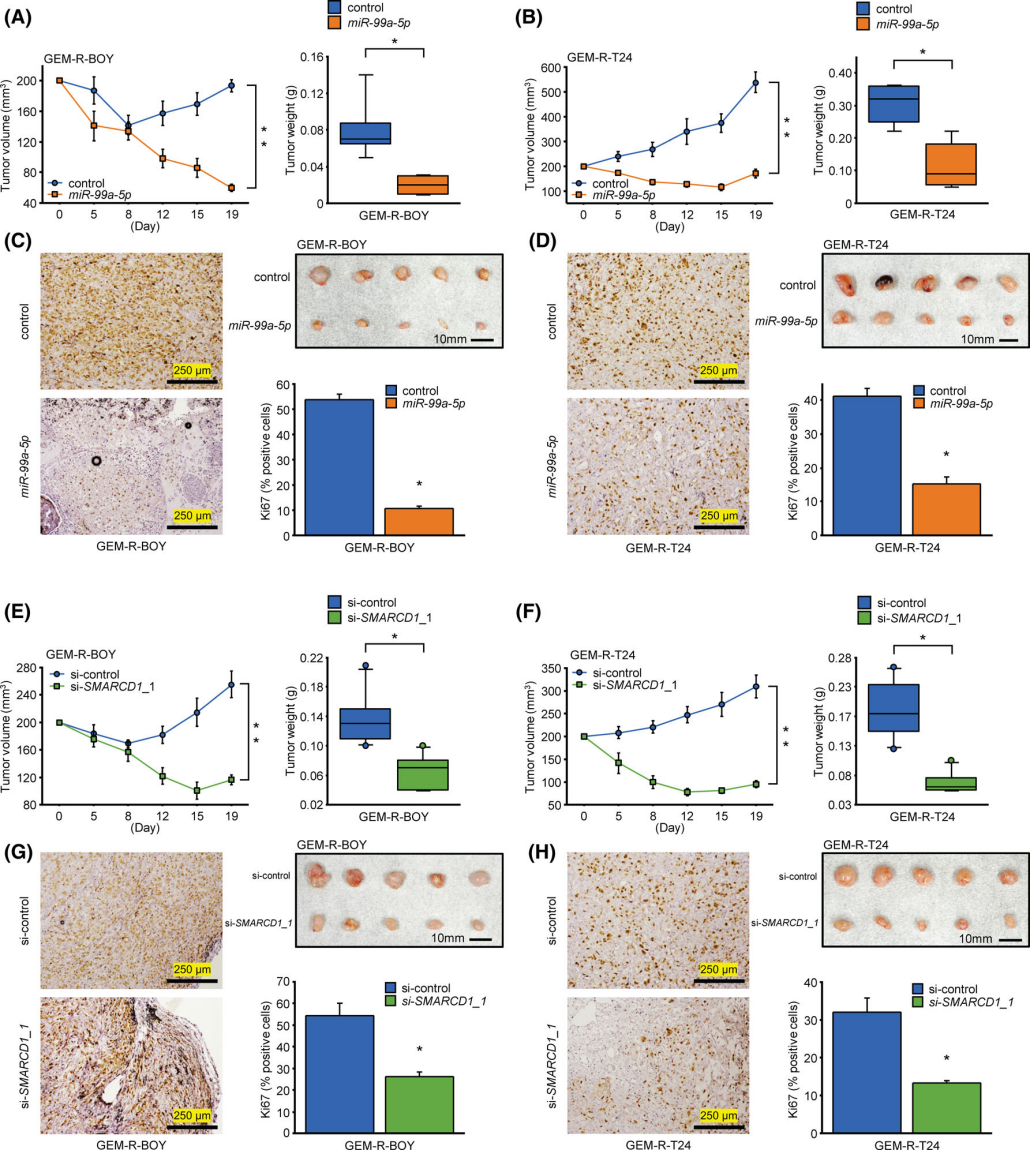
Overexpression of *miR‐99a‐5p* and knockdown of *SMARCD1* inhibit tumor growth *in vivo*. After transfection in GEM‐R BC cells with *miR‐99a‐5p* or si‐*SMARCD1*, the cells were injected into the flanks of nude mice (*n* = 5 mice per group). The animals with tumors were sacrificed in experimental observation for 19 days, and tumors were removed and weighed. Comparison of tumor volume trends and explant weights of (A) GEM‐R‐BOY cells and (B) GEM‐R‐T24 cells transfected with *miR‐99a‐5p*. *P < 0.01; **P < 0.0001, Mann–Whitney *U* tests. The error bars indicate SEM. Photograph shows excised tissue in the Xenograft mouse model on day 19. Scale bar, 10 mm. Ki67 expression in the xenograft tumors transfected with *miR‐99a‐5p* was detected by immunostaining. Micrographs of (C) GEM‐R‐BOY cells and (D) GEM‐R‐T24 cells (DAB, 100×). Positive cells were counted and compared. *n* = 6. *P < 0.05, Mann–Whitney *U* tests. The error bars indicate SEM. Scale bar, 250 μm. Comparison of tumor volume trends and explant weights of (E) GEM‐R‐BOY cells and (F) GEM‐R‐T24 cells transfected with si‐*SMARCD1*. *P < 0.01. **P < 0.0001, Mann–Whitney *U* tests. The error bars indicate SEM. Photograph shows excised tissue in the Xenograft mouse model on day 19. Scale bar, 10 mm. Ki67 expression in the xenograft tumors transfected with si‐*SMARCD1* was detected by immunostaining. Micrographs of (G) GEM‐R‐BOY cells and (H) GEM‐R‐T24 cells (DAB, 100×). Positive cells were counted and compared. *n* = 6. *P < 0.05, Mann–Whitney *U* tests. The error bars indicate SEM. Scale bar, 250 μm. GEM‐R BC, gemcitabine‐resistant bladder cancer.

Having confirmed *in vitro* studies that *miR‐99a‐5p* overexpression and *SMARCD1* knockdown affect cell function, we further investigated their effects on BC *in vivo*. First, GEM‐R BC cells (GEM‐R‐BOY, GEM‐R‐T24) transfected with miR‐NC or *miR‐99a‐5p* were injected subcutaneously into nude mice. Considering the duration of transfection, we observed the tumors for a short period of time within 20 days. The body weight and tumor diameter of nude mice were measured twice a week after inoculation. The results showed that overexpression of *miR‐99a‐5p* significantly suppressed tumor weight and volume *in vivo* (Fig. 5A,B). Immunostaining of excised tissues also showed decreased expression of Ki67, which reflects cell proliferation (Fig. 5C,D). Similarly, we examined the effect of siRNA on *SMARCD1*. si‐NC and si‐*SMARCD1*_1 transfected GEM‐R BC cells (GEM‐R‐BOY, GEM‐R‐T24) were injected subcutaneously into nude mice. The results showed that knockdown of *SMARCD1* significantly suppressed tumor weight and volume *in vivo* (Fig. 5E,F). Similarly, immunostaining of the excised tissue showed decreased expression of Ki67, which indicates cell proliferation (Fig. 5G,H). These data suggest that overexpression of *miR‐99a‐5p* and downregulation of *SMARCD1* in BC cells effectively inhibit the formation and growth of bladder cancer *in vivo*.

### 3.6 Relationship between *SMARCD1* and cellular senescence in GEM‐R BC cell lines

**Fig. 6:**
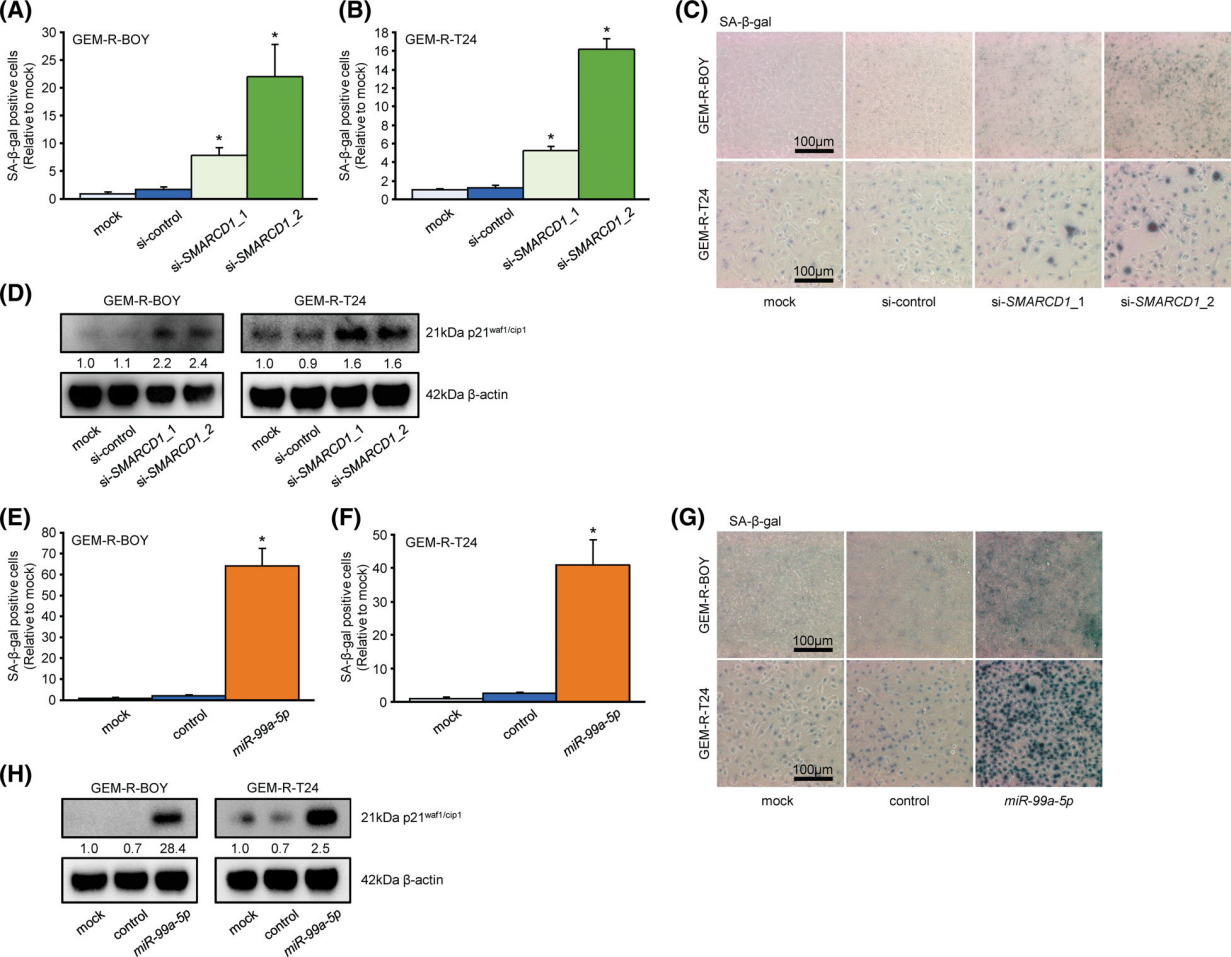
Relationship between *SMARCD1* and cellular senescence in the GEM‐R BC cell line. Senescence induction by si‐*SMARCD1* transfection was confirmed by β‐galactosidase staining. Quantification of β‐galactosidase staining in (A) GEM‐R‐BOY cells and (B) GEM‐R‐T24 cells. *n* = 6. *P < 0.005, Bonferroni/Dun method. The error bars indicate SEM. (C) A typical micrograph of (A) and (B). Scale bar, 100 μm. (D) Western blot confirming the expression of p21^waf1/cip1^ in GEM‐R BC cells transfected with si‐*SMARCD1*. Quantification of β‐galactosidase staining of (E) GEM‐R‐BOY cells and (F) GEM‐R‐T24 cells in *miR‐99a‐5p* transfection. *n* = 6. *P < 0.0001, Bonferroni/Dun method. The error bars indicate SEM. (G) A typical micrograph of (E) and (F). Scale bar, 100 μm. (H) Western blot confirming the expression of p21^waf1/cip1^ in GEM‐R BC cells transfected with *miR‐99a‐5p*. imagej was used for protein levels and quantification of β‐galactosidase staining. These experiments were repeated at least three times. GEM‐R BC, gemcitabine‐resistant bladder cancer; SA‐β‐gal, senescence‐associated beta‐galactosidase.

Since gemcitabine eventually induces apoptosis in cancer cells, we confirmed the expression of cleaved PARP by western blotting. We found that *miR‐99a‐5p* overexpression and *SMARCD1* knockdown in the GEM‐R BC cell line did not show a consistent increase in cleaved PARP expression compared to the target cells. However, *SMARCD1* knockdown showed increased expression of truncated PARP with low‐dose GEM stimulation (Fig. S5A). This result was also observed in apoptosis assays using flow cytometry (Fig. S5B), suggesting that *SMARCD1* knockdown may affect the proliferative capacity of GEM‐R BC cell lines by other mechanisms and may be involved in the sensitivity to gemcitabine. It has been reported that gemcitabine sensitivity in pancreatic cancer is improved by induction of cellular senescence [30], and *SMARCD1* has been reported to be involved in cellular senescence in hepatocytes [31]. Therefore, we examined the involvement of *SMARCD1* in senescence in the GEM‐R BC cell line. We found that knockdown of *SMARCD1* induced senescence‐associated β‐galactosidase (SA‐β‐gal) activity (Fig. 6A–C) and upregulated the expression of p21^waf1/cip1^ in western blots (Fig. 6D). Similarly, transfection of *miR‐99a‐5p* also showed these changes (Fig. 6E–H, Fig. S5C,D), suggesting that low expression of *SMARCD1* and overexpression of *miR‐99a‐5p* may be involved in the induction of cellular senescence, affecting cell proliferation, migration, invasion, and sensitivity to gemcitabine.

## 4 Discussion

miRNAs are considered to be important regulators of cell proliferation, differentiation, development, and apoptosis in cancer cells [32, 33]. We have shown that certain miRNAs are aberrantly expressed and target several oncogenes and pathways, thereby affecting cancer progression [7, 8, 34, 35, 36]. In addition, miRNAs are involved in drug resistance, as we previously reported that *miR‐486‐5p* regulates *EHHADH* in BC and contributes to cisplatin resistance [11]. Regarding gemcitabine resistance, Yang et al. reported that *miR‐760* improves gemcitabine resistance in pancreatic cancer by regulating ITGB1 stabilized by MOV10 [17]. In addition, Zhang et al. [18] reported that *miR‐205‐5p* is involved in gemcitabine resistance by targeting PRKCE in gallbladder cancer. In this study, we focused on *miR‐99a‐5p*. We observed that its expression was downregulated in GEM‐R BC cell lines (GEM‐R‐BOY, GEM‐R‐T24) compared to the parental BC cell lines (BOY, T24). Interestingly, *miR‐99a* is frequently downregulated in other types of cancers, such as breast cancer, cholangiocarcinoma, esophageal cancer, and squamous cell carcinoma of the head and neck. There are also reports that *miR‐99a* is potentially involved in tumorigenesis of many types of cancers [37] as well as drug resistance. Sun et al. reported that circMCTP2 improves cisplatin resistance by inhibiting *miR‐99a‐5p* in gastric cancer [29], and Dhayat et al. reported increased expression of several miRNAs including *miR‐99a* in gemcitabine‐resistant pancreatic cancer [38]. Therefore, our results that *miR‐99a* was associated with gemcitabine are consistent with other studies. Furthermore, changes in radio‐sensitivity in non‐small cell lung cancer have been observed [39].

*SMARCD1*, which is a member of the SWI/SNF chromatin remodeling complex family, regulates the transcription of target genes through changes in chromatin structure [40]. For example, Rane et al. [41] reported that *SMARCD1* was targeted by *miR‐99a* in prostate cancer. We first demonstrated that *SMARCD1* was directly regulated by *miR‐99a‐5p*. In addition, we showed that the level of SMARCD1 protein was increased in the GEM‐R BC cell line compared to the parental cell line. To the best of our knowledge, there have been no reports on the relationship between *SMARCD1* and drug resistance. On the other hand, Akrami et al. [42] reported that in gastric cancer, ibuprofen was involved in the altered expression of multiple genes, including *SMARCD1* and that it reduced cell proliferation by inhibiting the Wnt/β‐catenin signaling pathway. In this study, we also confirmed that knockdown of the *SMARCD1* gene had a significant inhibitory effect on cancer cell proliferation, migration, and invasion, and also improved cell sensitivity to gemcitabine. However, there was no significant correlation between *SMARCD1* expression level and history of treatment with gemcitabine in the TCGA database. This may be due to the limited sample size of TCGA database and the fact that advanced bladder cancer is not usually treated with gemcitabine alone, or may have been preceded by chemotherapy with other drugs. Further study is necessary to elucidate the mechanism of *SMARCD1* to gemcitabine resistance. In this study, we also demonstrated that transient induction of *miR‐99a‐5p* and knockdown of *SMARCD1* affect cell proliferative capacity *in vivo* and confirmed that Ki‐67‐positive cells were reduced in those tumor fractions compared with controls. However, we only performed *in vitro* experiments regarding migration and invasion in this study, it is also necessary to elucidate whether *miR‐99a‐5p* and *SMARCD1* affects metastasis *in vivo*, and identify other potential target genes which were related with metastasis in the future study.

Cellular senescence exhibits a stable and prolonged loss of proliferative capacity, despite continued viability and metabolic activity [43]. In addition, various stresses induce a similar phenotype referred to as premature senescence. In particular, aging induced by the activation of oncogenes such as the *RAS* gene is called oncogene‐induced senescence (OIS). OIS is thought to function as a carcinogenesis defense mechanism [44]. On the other hand, cellular senescence and OIS develop a senescence‐associated secretory phenotype (SASP) in which secreted bioactive substances (cytokines) affect the behavior of neighboring cells [45]. Recently, it was reported that cellular senescence was associated with chemotherapy resistance regardless of gemcitabine [46]. Schmitt et al. [47] reported that tumor senescence affected the therapeutic effect of chemotherapy in mice. Many SASP factors are known to stimulate oncogenic induction, while others reportedly have anticancer effects. Ruscetti et al. [30] reported that by causing cellular senescence in pancreatic cancer, SASP causes angiogenesis and immune cell activation, which increases the efficacy of gemcitabine and immune checkpoint inhibitors. Song et al. [48] reported that gemcitabine induced senescence in pancreatic cancer and other researchers have reported that gemcitabine induces senescence in cancer cells. Cellular senescence induced by chemotherapy and radiotherapy is called therapy‐induced senescence (TIS) [49]. On the other hand, there is a report that the proliferative capacity of cancer stem cells increases if they survive aging [50]. We suggest that the enhanced cellular functions of established GEM‐R BC cell lines, such as proliferation, are a result of the senescence induced in GEM‐R BC strains by exposure to gemcitabine and their eventual avoidance of senescence. In addition, *SMARCD1* is involved in senescence. Inoue et al. [31] reported that low expression of *SMARCD1* induces senescence in hepatocytes. In the present study, we confirmed that knockdown of *SMARCD1* and overexpression of *miR‐99a‐5p* induced senescence in bladder cancer cell lines. Based on the aforementioned reports, senescence induction by *SMARCD1* knockdown is plausible due to its relationship with *miR‐99a‐5p*. However, senescence induction has not been reported previously and must be considered novel. On the other hand, whereas *SMARCD1* knockdown and *miR‐99a‐5p* overexpression do not induce apoptosis by themselves, they can induce apoptosis by improving the sensitivity to gemcitabine. How this senescence mechanism improves gemcitabine tolerance and enhances the induction of apoptosis in the absence of blood vessels and immune cells is unclear. Further studies are needed to elucidate the involvement of *SMARCD1* in gemcitabine tolerance through senescence.

## 5 Conclusion

The expression level of *miR‐99a‐5p* was decreased in GEM‐R BC cells. Overexpression of *miR‐99a‐5p* induced cellular senescence, significantly inhibited cancer cell proliferation and restored sensitivity to gemcitabine by directly regulating *SMARCD1*. The identification of *miR‐99a‐5p* as a key molecule for overcoming gemcitabine resistance may lead to a better understanding of BC and the development of new therapeutic strategies for this disease.

## Conflict of interest

The authors declare no conflict of interest.

## Author contributions

MT designed the study, collected and analyzed data, and wrote the initial draft of the manuscript; WF, IK, SO, YO, TS, and SS performed experiments and collected and analyzed data; HY, MY, YY, MN, and HE secured research funding and assisted in the preparation of the manuscript. All authors have contributed to data interpretation and critically reviewed the manuscript and approved the final version of the manuscript, and consent to be accountable for all aspects of the work in ensuring that questions related to the accuracy or integrity of any part of the work are appropriately investigated and resolved.

### Peer review

The peer review history for this article is available at https://publons.com/publon/10.1002/1878‐0261.13192.

## Supporting information


**Fig. S1**. Comparison of the parental BC cell line with the GEM‐R BC cell line.
**Fig. S2**. Comparison of mock and miR‐control transfectants with *miR‐99a‐5p* transfectants.
**Fig. S3**. mRNA expression levels in BC and GEM‐R BC cell lines, and *SMARCD1* and *miR‐99a‐5p* expression levels in BC specimens.
**Fig. S4**. Comparison of mock or si‐control transfectants with si‐*SMARCD1* transfectants.
**Fig. S5**. Confirmation of apoptosis by western blot and flow cytometry.

## Data Availability

The data that support the findings of this study are openly available in figshare. The reference numbers are as follows: https://doi.org/10.6084/m9.figshare.17698598, https://doi.org/10.6084/m9.figshare.17698607, https://doi.org/10.6084/m9.figshare.17698529, https://doi.org/10.6084/m9.figshare.17698592.
