# Supplementary material for: microRNA‐99a‐5p induces cellular senescence in gemcitabine‐resistant bladder cancer by targeting SMARCD1
Source: Mol Oncol. 2022 Feb 28;16(6):1329–46. doi: 10.1002/1878-0261.13192 (PMC8936529; doi:10.1002/1878-0261.13192)
Supplement: Supplementary file 1 — Fig. S1. Comparison of the parental BC cell line with the GEM‐R BC cell line. Fig. S2. Comparison of mock and miR‐control transfectants with miR‐99a‐5p transfectants. Fig. S3. mRNA expression levels in BC and GEM‐R BC cell lines, and SMARCD1 and miR‐99a‐5p expression levels in BC specimens. Fig. S4. Comparison of mock or si‐control transfectants with si‐SMARCD1 transfectants. Fig. S5. Confirmation of apoptosis by western blot and flow cytometry. [file MOL2-16-1329-s001.pdf]

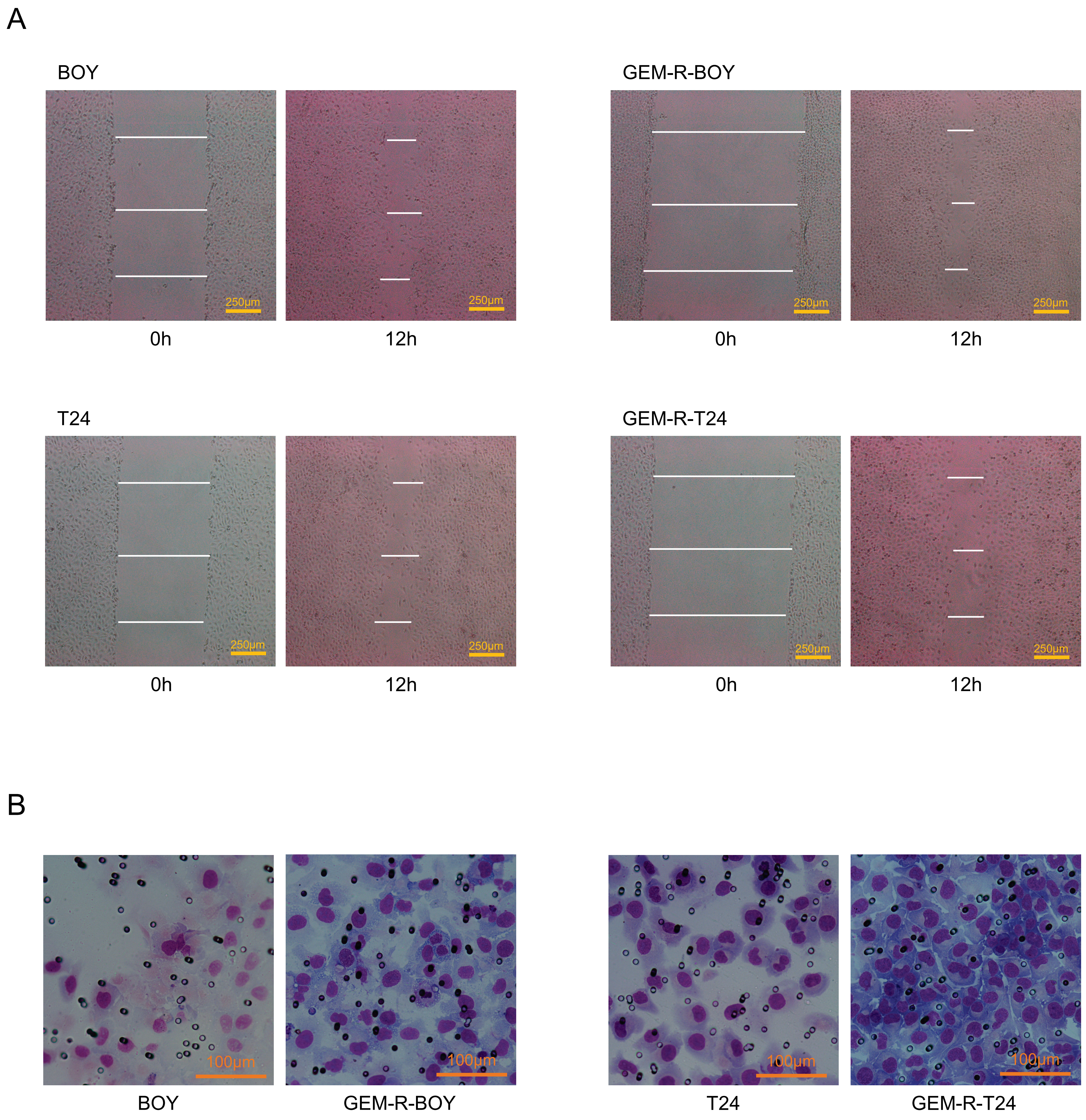

Supplementary Figure 1. Comparison of the parental BC cell line with the GEM-R BC cell line. (A) Representative pictures of cell migration assay. Scale bar, 250 µm. (B) Representative pictures of cell invasion assay. Scale bar, 100 µm. These experiments were repeated at least three times.

Supplementary Figure2

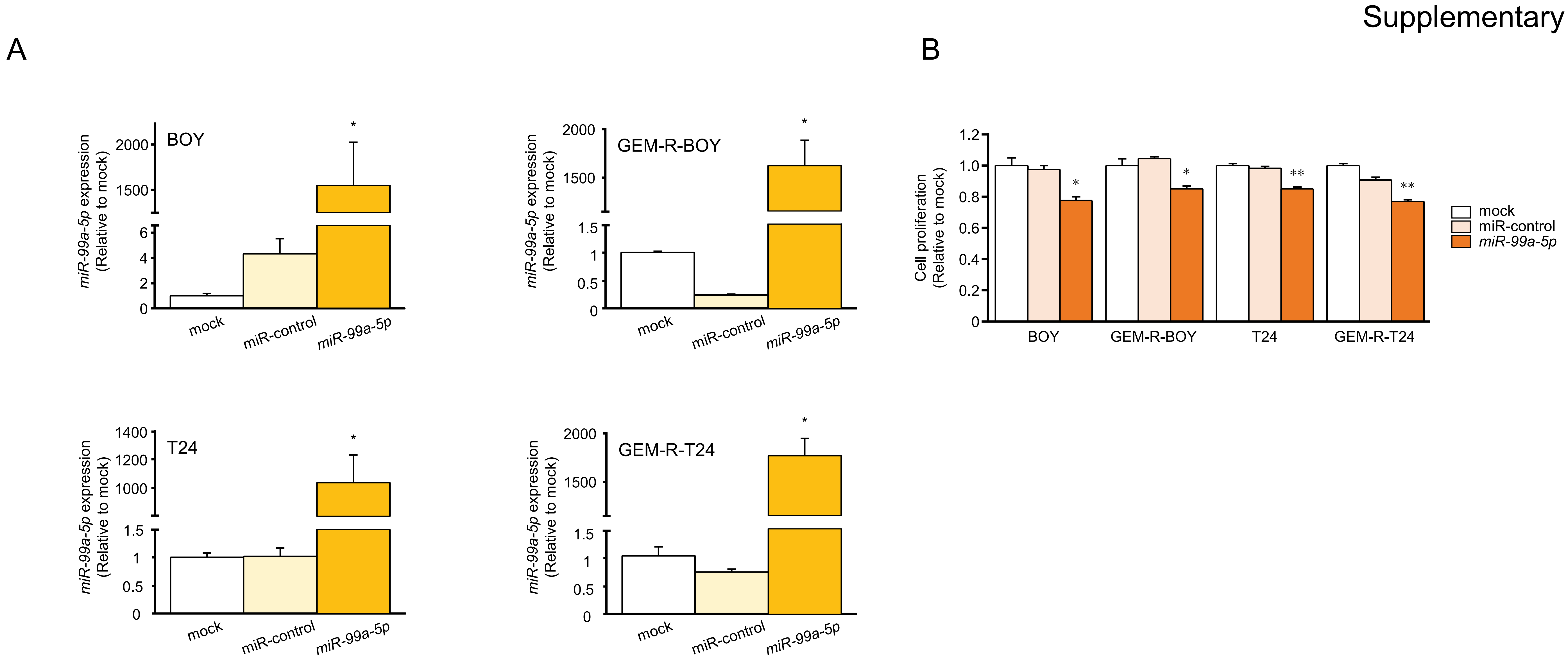

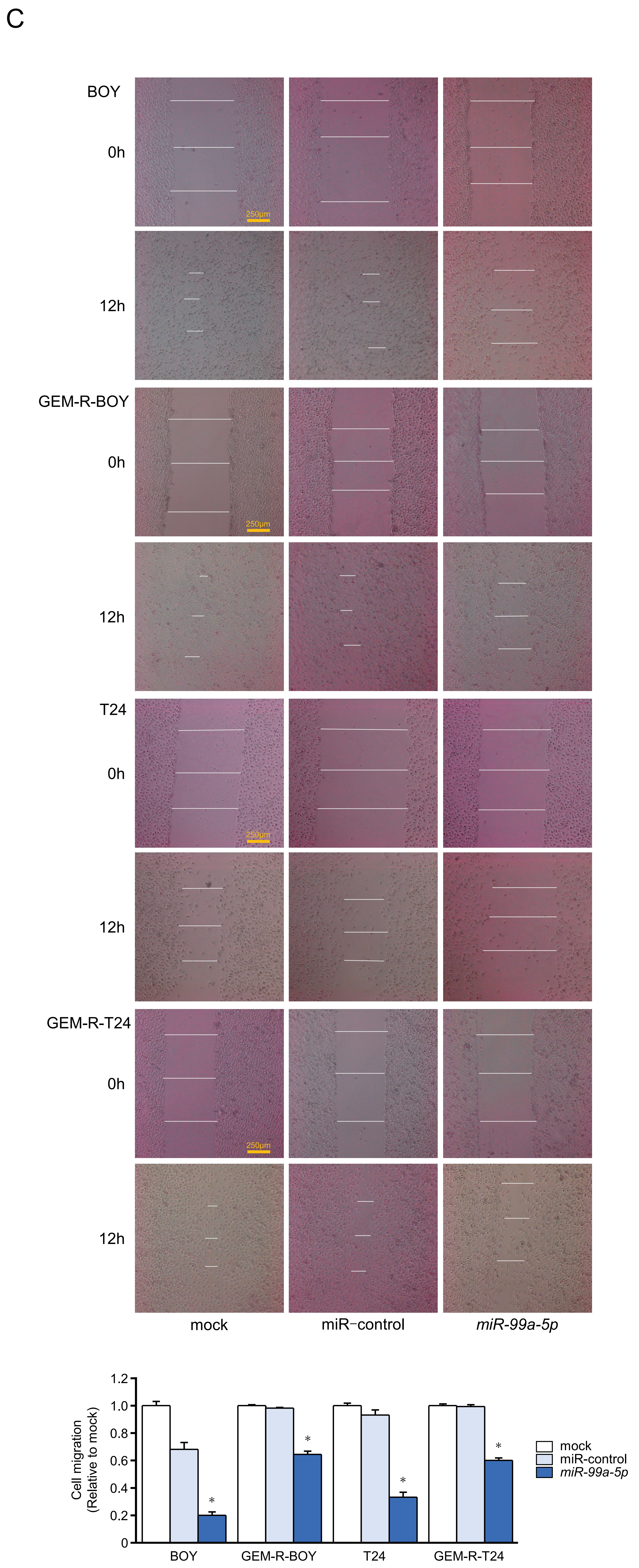

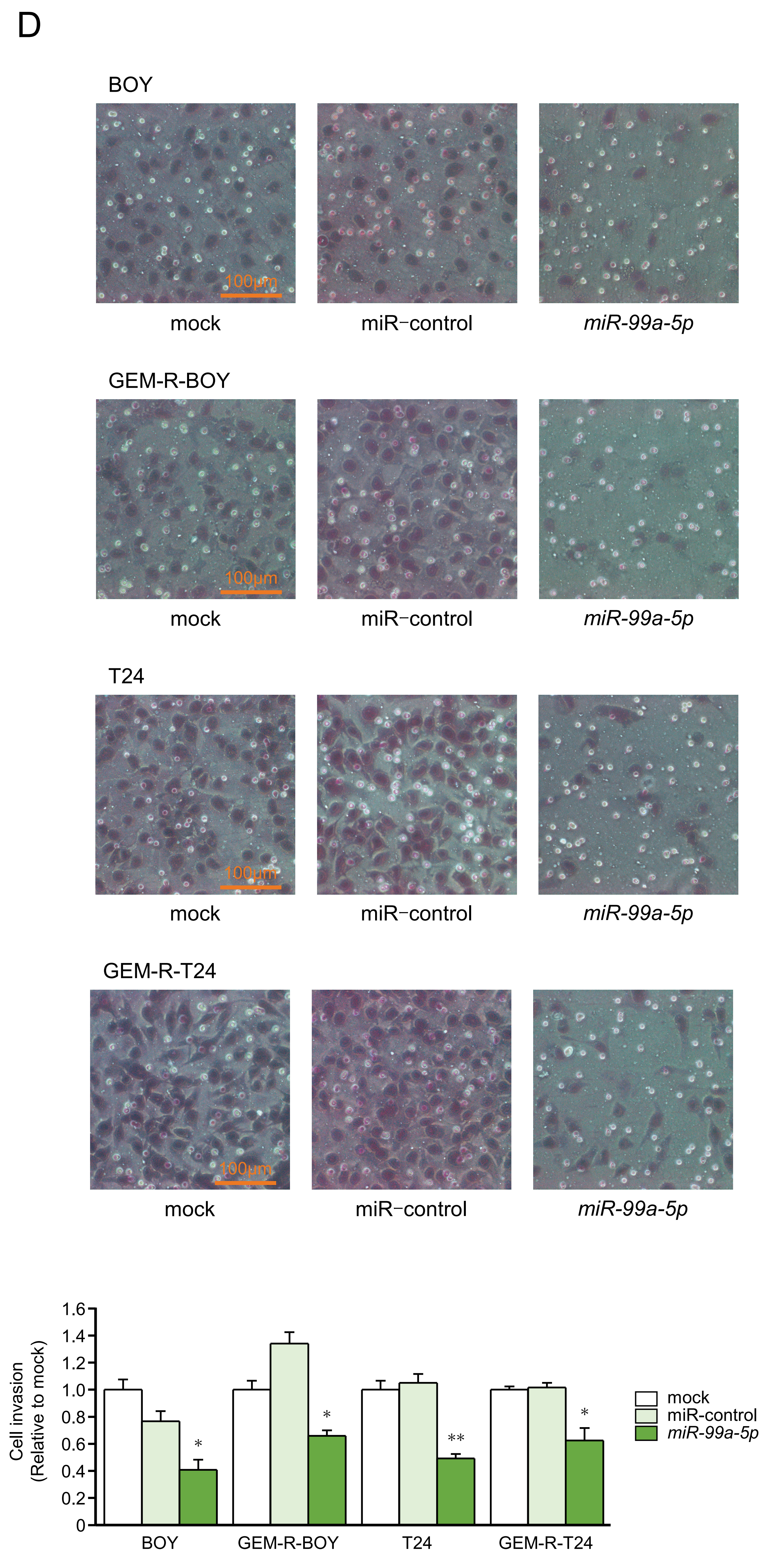

Supplementary Figure 2. Comparison of mock and miR-control transfectants with *miR-99a-5p* transfectants. (A) Expression levels of *miR-99a-5p* quantified by qRT-PCR. *, P < 0.05. (B) Cell proliferation measured by XTT assay. Comparison was made for each cell type with relative to mock. *, P < 0.01. **, P < 0.001. (C) Representative pictures of cell migration assay. Scale bar, 250 μm. Comparison was made for each cell type with relative to mock. *, P < 0.0001. (D) Representative pictures of cell invasion assay. Scale bar, 100 μm. Comparison was made for each cell type with relative to mock. Invasion cells were counted and compared. *, P < 0.01. **, P < 0.001. These experiments were repeated at least three times.

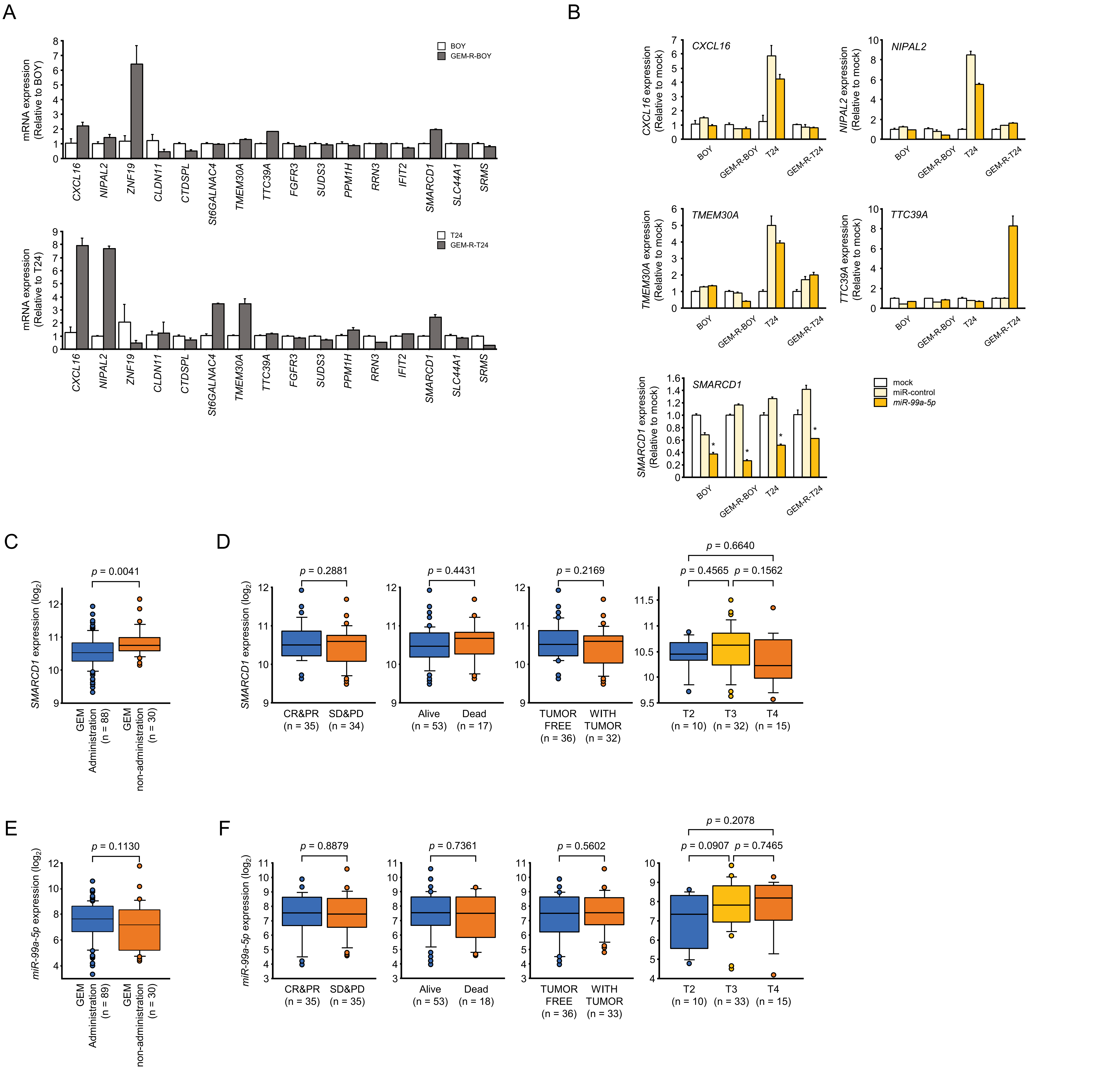

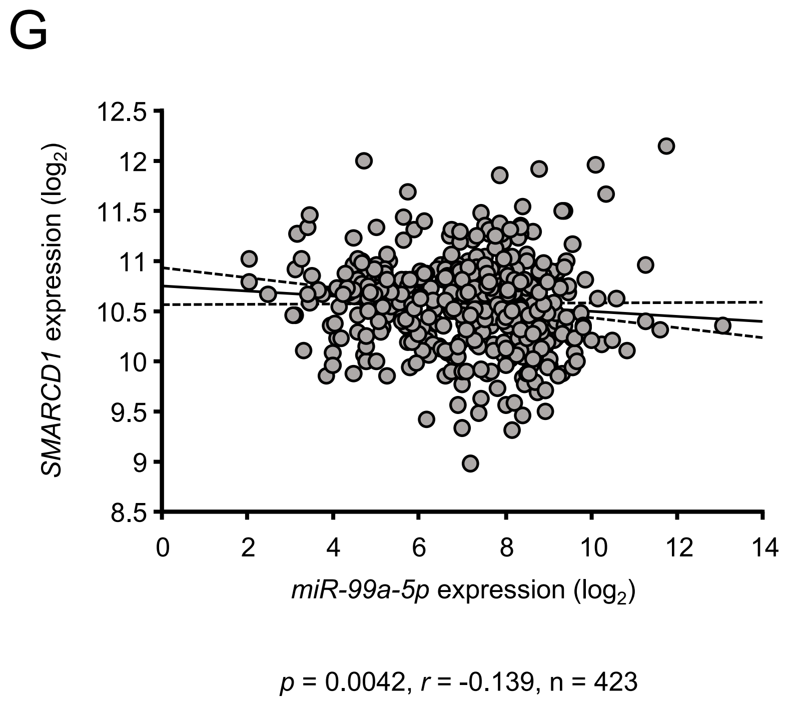

Supplementary Figure 3. mRNA expression levels in BC and GEM-R BC cell lines, and *SMARCD1* and *miR-99a-5p* expression levels in BC specimens. (A) Comparison of the expression levels of candidate genes determined by qRT-PCR analysis between parental BC cells and GEM-R BC cells. Among the candidates, five genes, *CXCL16*, *NIPAL2*, *TMEM30A*, *TTC39A* and *SMARCD1*, were upregulated in GEM-R BC cells. (B) Comparison of the expression levels of the five candidate genes by qRT-PCR between mock or miR-control transfectants and *miR-99a-5p* transfectants in parental BC cells and GEM-R BC cells. *SMARCD1* showed uniform downregulation in *miR-99a-5p* transfectants. *, P < 0.0001. (C) Expression of *SMARCD1* in bladder cancer samples from TCGA database. When the relationship with the presence or absence of gemcitabine administration history was investigated, a decrease in expression was observed in the group with administration history. (D) Expression of *SMARCD1* in bladder cancer samples from TCGA database. For patients who had been treated with gemcitabine, a comparison of cohorts including CR (Complete Response) and PR (Partial Response) patients and a cohort including SD (Stable Disease) and PD (Progressive Disease) patients who did not have tumor suppression, a comparison by life and death after treatment, a comparison by presence or absence of tumors after treatment, a comparison of clinical pathological parameters (T classification). No significant difference was found in any of them. (E) Expression levels of *miR-99a-5p* in bladder cancer samples from TCGA database. The relationship with the history of gemcitabine administration was examined, and no significant difference was found. (F) Expression levels of *miR-99a-5p* in bladder cancer samples from TCGA database. For patients who had been treated with gemcitabine, a comparison of cohorts including CR (Complete Response) and PR (Partial Response) patients and a cohort including SD (Stable Disease) and PD (Progressive Disease) patients who did not have tumor suppression, a comparison by life and death after treatment, a comparison by presence or absence of tumors after treatment, a comparison of clinical pathological parameters (T classification). No significant difference was found in any of them. (G) Correlation of *miR-99a-5p* and *SMARCD1* expression levels in bladder cancer samples from TCGA database. Spearman's rank tests were used to evaluate the correlations.

A

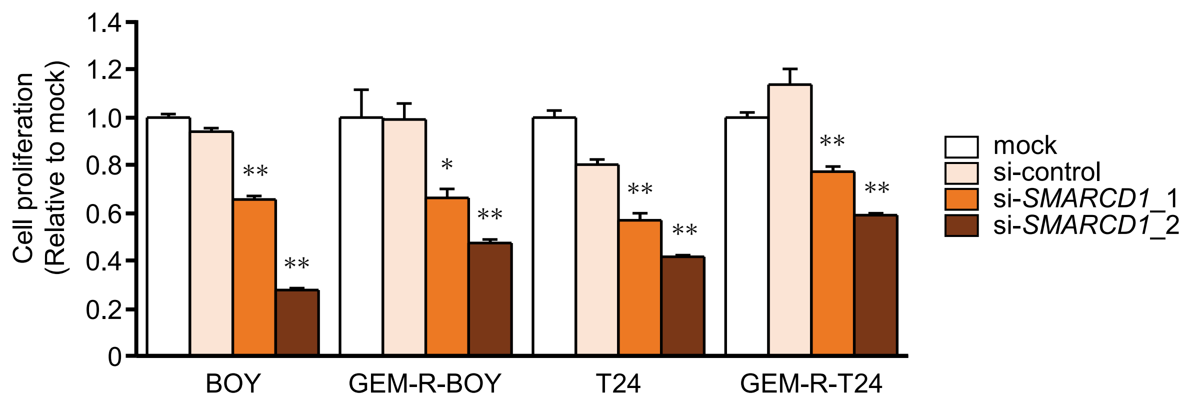

B

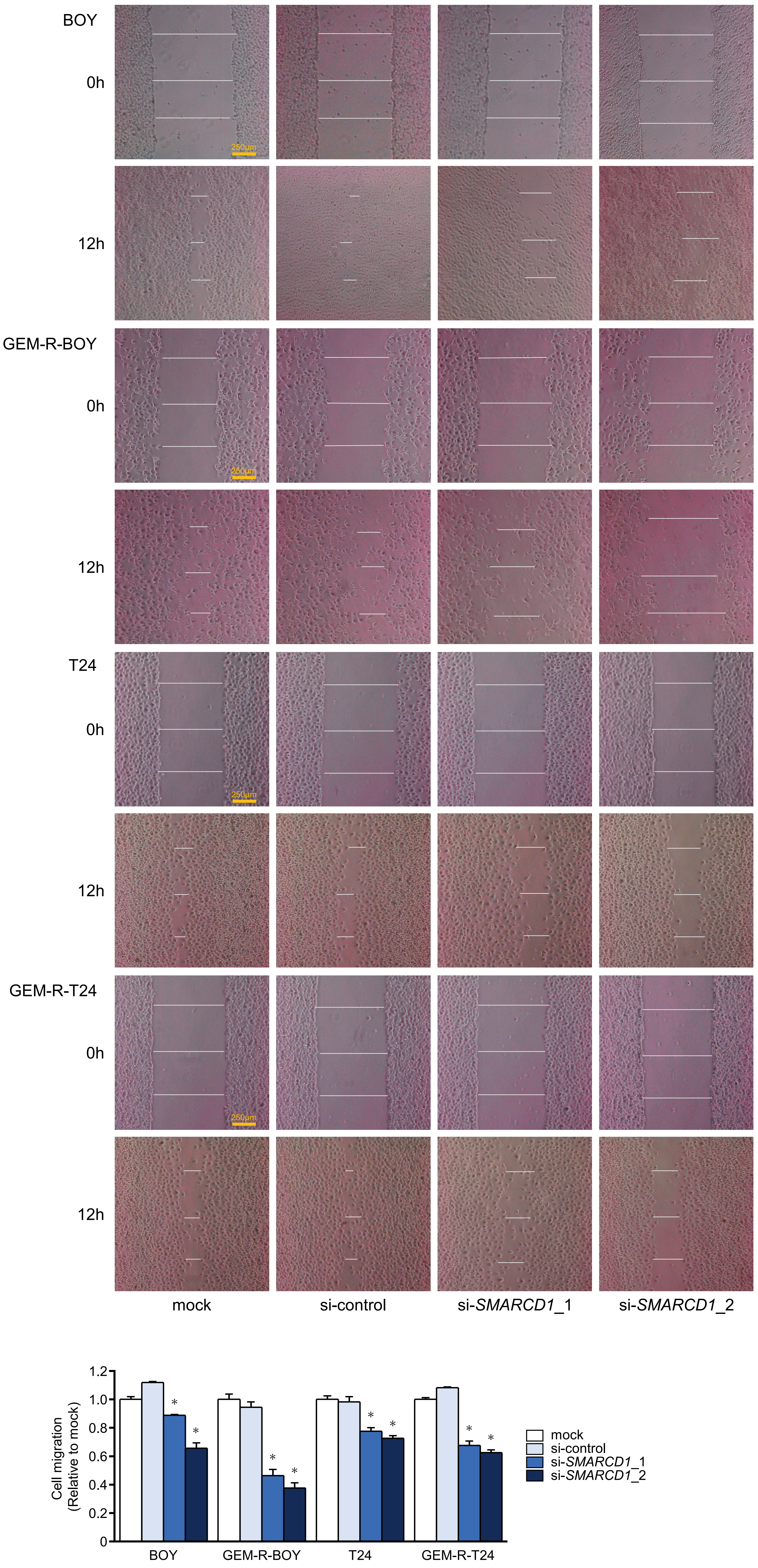

C

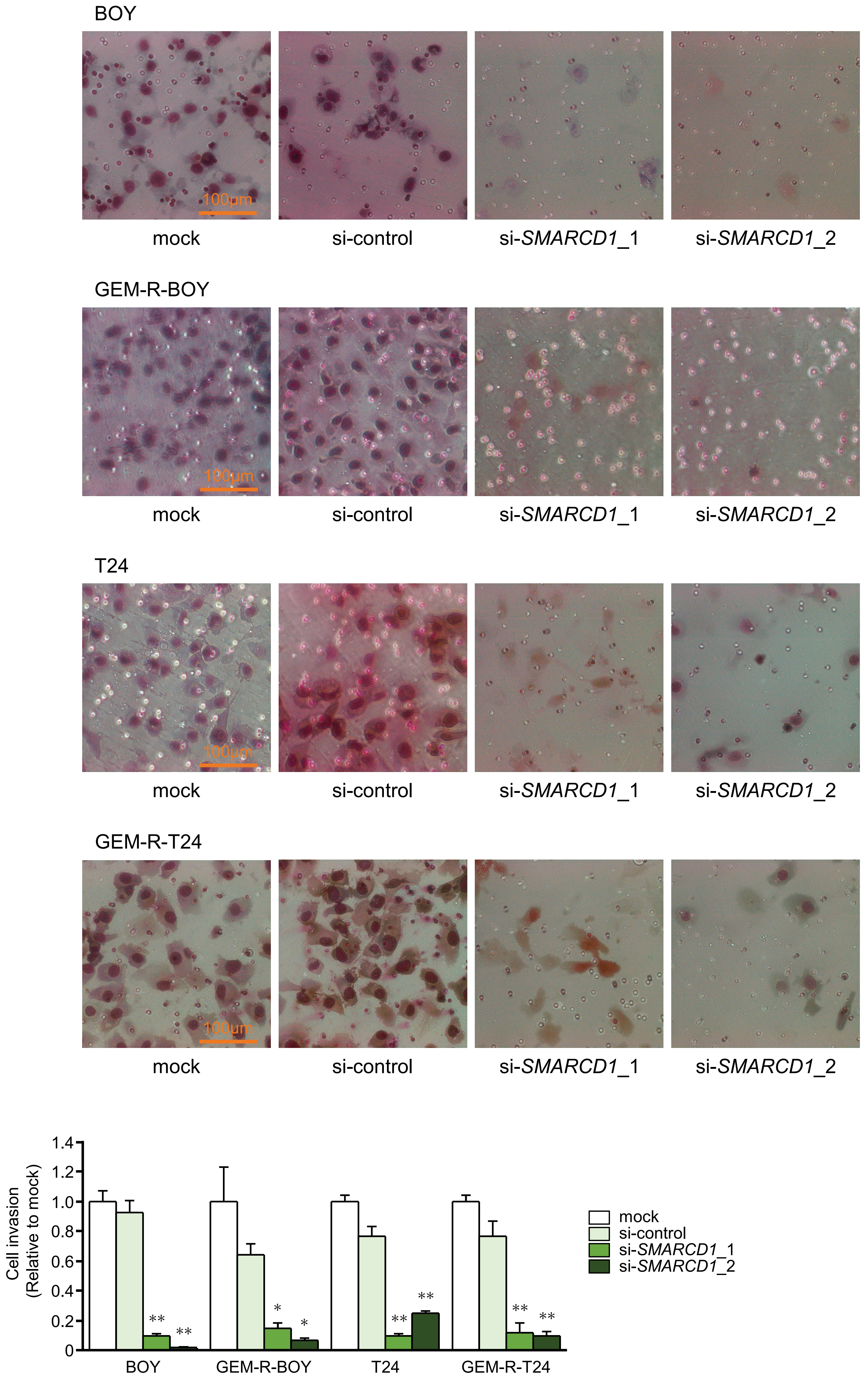

Supplementary Figure 4. Comparison of mock or si-control transfectants with si-*SMARCD1* transfectants. (A) Cell proliferation measured by XTT assay. Comparison was made for each cell type with relative to mock. *, P < 0.001. **, P < 0.0001. (B) Representative pictures of cell migration assay. Scale bar, 250 µm. Comparison was made for each cell type with relative to mock. *, P < 0.0001. (C) Representative photograph of cell invasion assay. Scale bar, 100 µm. Comparison was made for each cell type with relative to mock. Invasion cells were counted and compared. *, P < 0.001. **, P < 0.0001. These experiments were repeated at least three times.

Supplementary Figure5

A

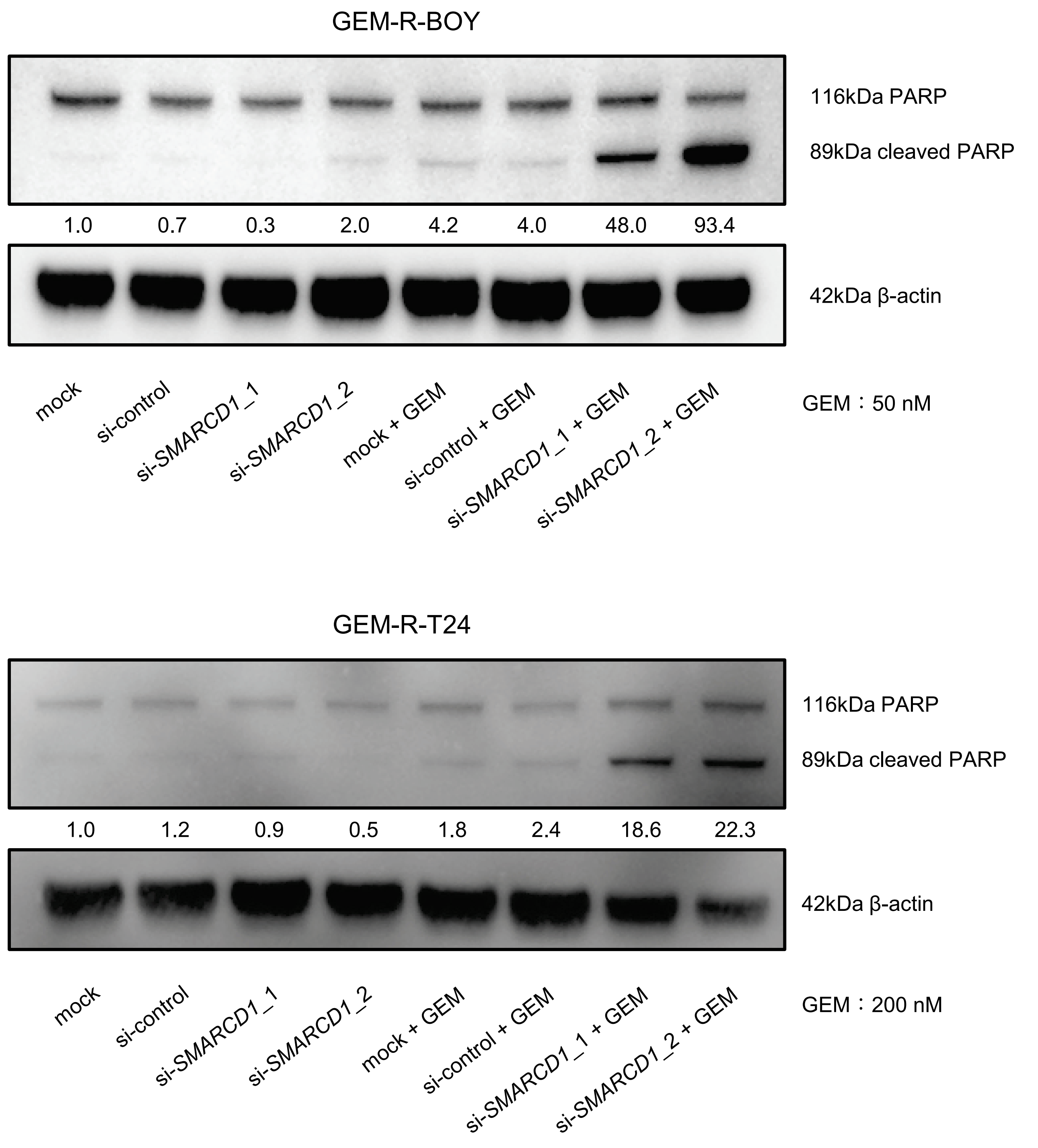

B

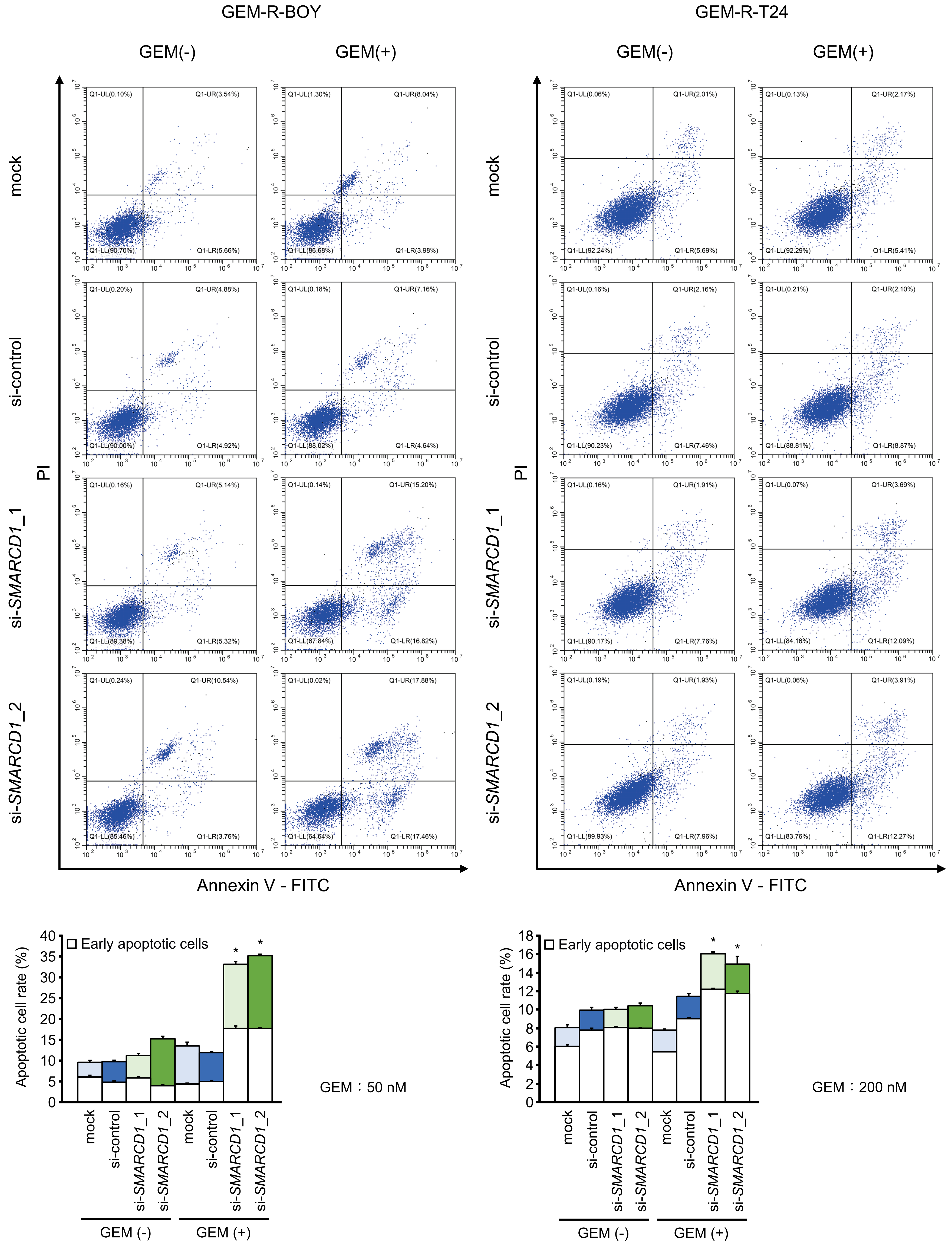

C

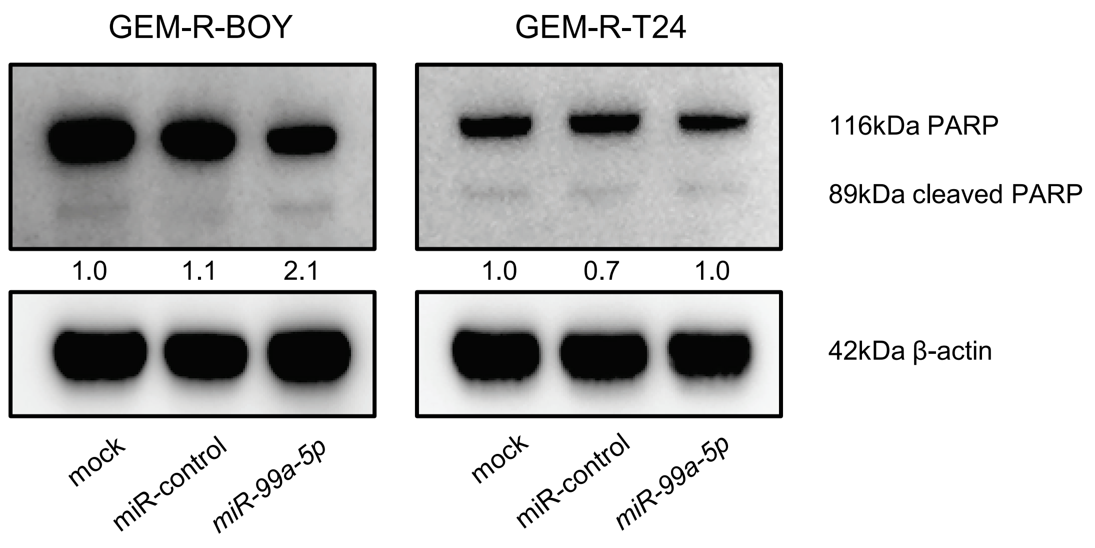

D

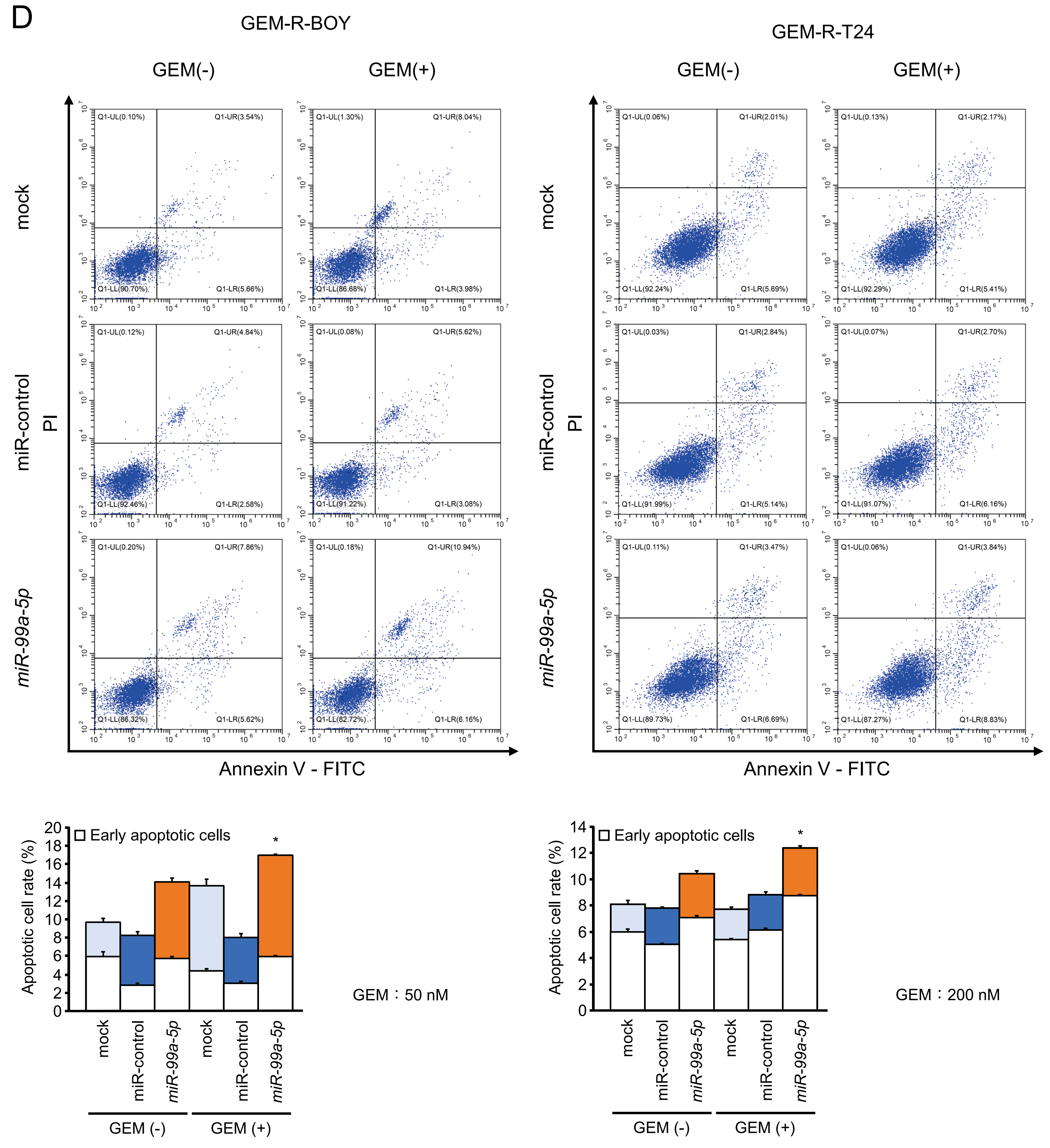

Supplementary Figure 5. Confirmation of apoptosis by Western blot and flow cytometry. (A) Comparison of mock or si-control transfectants and si-*SMARCD1* transfectants. Expression of cleaved PARP in Western blot. ImageJ was used for protein levels. (B) Apoptosis assessed by flow cytometry. The ratio of total apoptotic cells to early apoptotic cells. si-*SMARCD1* transfection in combination with a small amount of gemcitabine resulted in a significantly higher number of apoptotic cells than did treatment alone. *, P < 0.0001. (C) Comparison of mock or miR-control transfectants with *miR-99a-5p* transfectants. Expression of cleaved PARP in Western blot. ImageJ was used for protein levels. (D) Apoptosis assessed with flow cytometry. Ratio of total apoptotic cells to early apoptotic cells. *miR-99a-5p* transfection in combination with a small amount of gemcitabine resulted in a significantly higher number of apoptotic cells than did treatment alone. *, P < 0.005. These experiments were repeated at least three times.
